# Integrated analysis of robust sex-biased gene signatures in human brain

**DOI:** 10.1186/s13293-023-00515-w

**Published:** 2023-05-24

**Authors:** Pattama Wapeesittipan, Anagha Joshi

**Affiliations:** Department of Clinical Sciences, Computational Biology Unit, University of Bergen, Bergen, Norway

**Keywords:** Sex difference, Human brain, Gene regulation, Hormones, Data integration, Conservation, Brain disorders, Drug response

## Abstract

**Background:**

Sexual dimorphism is highly prominent in mammals with many physiological and behavioral differences between male and female form of the species. Accordingly, the fundamental social and cultural stratification factors for humans is sex. The sex differences are thought to emerge from a combination of genetic and environmental factors. It distinguishes individuals most prominently on the reproductive traits, but also affects many of the other related traits and manifest in different disease susceptibilities and treatment responses across sexes. Sex differences in brain have raised a lot of controversy due to small and sometimes contradictory sex-specific effects. Many studies have been published to identify sex-biased genes in one or several brain regions, but the assessment of the robustness of these studies is missing. We therefore collected huge amount of publicly available transcriptomic data to first estimate whether consistent sex differences exist and further explore their likely origin and functional significance.

**Results and conclusion:**

In order to systematically characterise sex-specific differences across human brain regions, we collected transcription profiles for more than 16,000 samples from 46 datasets across 11 brain regions. By systematic integration of the data from multiple studies, we identified robust transcription level differences in human brain across to identify male-biased and female-biased genes in each brain region. Firstly, both male and female-biased genes were highly conserved across primates and showed a high overlap with sex-biased genes in other species. Female-biased genes were enriched for neuron-associated processes while male-biased genes were enriched for membranes and nuclear structures. Male-biased genes were enriched on the Y chromosome while female-biased genes were enriched on the X chromosome, which included X chromosome inactivation escapees explaining the origins of some sex differences. Male-biased genes were enriched for mitotic processes while female-biased genes were enriched for synaptic membrane and lumen. Finally, sex-biased genes were enriched for drug-targets and more female-biased genes were affected by adverse drug reactions than male-biased genes. In summary, by building a comprehensive resource of sex differences across human brain regions at gene expression level, we explored their likely origin and functional significance. We have also developed a web resource to make the entire analysis available for the scientific community for further exploration, available at https://joshiapps.cbu.uib.no/SRB_app/

**Supplementary Information:**

The online version contains supplementary material available at 10.1186/s13293-023-00515-w.

## Plain language summary

Sex and gender differences are present across many organs in humans and have biological and social origins. The differences in brain raise a lot controversy due to small and sometimes contradictory results and its societal implications. In this study, we set out to discern the consistency of sex differences in brain by collecting a huge amount of publicly available transcriptomic data and further explore their likely origin and functional significance. We identified robust sex-biased genes in human brain with female-biased genes enriched for X chromosome genes. We also noted that male- and female-biased genes were enriched for distinct biological processes. Finally, sex-biased genes were enriched for androgen response elements. In summary, our analysis suggests sex-chromosomes and androgens as likely sources of sex differences in brain. Finally, we noted that age affects gene expression in brain more than sex.

**Supplementary Information:**

The online version contains supplementary material available at 10.1186/s13293-023-00515-w.

## Highlights

- By collecting and reanalyzing 16,000 samples from 46 datasets, we identified robust male- and female-biased genes across 11 brain regions in humans. Female-biased genes were significantly enriched on the X chromosome and male-biased genes were enriched on the Y chromosome. Robust sex-biased genes were highly conserved across primates and other species.
- Sex-biased genes in the brain were enriched for brain-specific genes, but not region-specific genes within the brain. Male-biased genes across brain regions were enriched for astrocyte and oligodendrocyte signature genes more than female-biased genes.
- We noted that both age and sex influence gene expression for most genes. XIST was highly female-biased, while genes on the Y chromosome were male-biased. Three genes were found to be age- and sex-associated in both datasets, including FREM3, CHI3L1, and SERPINA3, which have been associated with neurological disease. Notably, CHI3L1 is highly expressed in female AD patients compared to male AD samples.
- Over 80% of both male and female-biased genes were enriched for either half or full androgen response element (ARE) sites across brain regions, and sex-biased genes are also enriched for estrogen response elements (ERE).
- Finally, we developed a web resource to make the entire analysis available for the scientific community for further exploration, available at https://joshiapps.cbu.uib.no/SRB_app/

**Supplementary Information:**

The online version contains supplementary material available at 10.1186/s13293-023-00515-w.

## Background

Biological sex is one of the most prominent stratification factor for the human population, with classical binary biological grouping into male and female. The physiological and behavioral sexual dimorphism in humans originates from both genetic and environmental constructs, and can produce divergent sex-specific disease susceptibility. For example, females carry a much higher burden of autoimmune diseases compared to men, while men are more likely to suffer from schizophrenia. Interestingly, the same alleles of the complement component 4 or C4 genes at the major histocompatibility complex (MHC) locus were shown to increase risk for schizophrenia and reduce risk for autoimmune disorders [1]. Sex and gender terms have been used inter-changeably in scientific literature. Sex is biologically determined by chromosomal makeup, while gender is more behavioral in nature and also more controversial as to how it is determined [2]. Importantly, most sexually dimorphic traits are likely to be a result of multiple, independent sex-biasing factors where genetic and epigenetic factors are manifested through sex-biased gene expression or hormonal control [3]. Such traits are defined as ’sex and gender’ or ’sex/gender terms’ or simply as ’sex’. Hence forth, we will use the term ’sex’ for simplicity.

Male and females have many differences, in physical appearance, social behavior as well as in disease incidence, prevalence, morbidity and mortality. Yet males have been predominantly used in basic and pre-clinical research, due to female cyclic hormonal patterns and importantly, a common belief that male and females mainly have only reproductive difference [4]. Historically clinical trials are largely conducted on males only and unsurprisingly, females are more likely to suffer from side effects from medications due to under-representation in clinical trials [5]. Despite this, scientific publications in pharmacology field show a trend downward with 29% of articles reporting the use of both sexes in 2019 compared to 33% in 2009 [6]. Studies of both males and females are essential to understanding sex-specific human biology towards the advancement of human health. There is growing scientific literature exploring sex differences in healthy lifespan and aging. Transcriptomic studies allow exploration of sex differences at genome-wide level providing clues for the molecular basis of sex differences. GTEX consortium generated transcriptome profiles across 53 human tissues using RNA-sequencing data for 544 individuals (males and females). Several studies have used this data to characterise sex differences across tissues [7–10]. Sex-specific differences are noted in all organs and these differences also affect tissues not specialized for reproduction, including non-reproductive tissues. Sex influences gene expression levels and cellular composition of tissue samples across the human body, with a total of 37% of all genes exhibiting sex-biased expression in at least one tissue [10].

Areas of the brain function differently in females and males, and are differentially affected by disease in the two sexes. For example, genes associated with Parkinson’s disease and Alzheimer’s disease are targeted by different sets of transcription factors in each sex [11]. Evaluating differences in male and female brains can contribute to understanding sex differences in disease incidence, manifestation, and outcome. Accordingly, several transcriptome studies have focused specifically on the human brain regions to identify sex-biased genes [12–14]. Sex differences in human brain have nevertheless remained controversial due to small effects and inconsistencies across studies as most of these studies have used mostly one or in rare cases a few [15] independent datasets making it hard to estimate the reproducibility of their sex-biased gene lists. Only a handful of studies have made a systematic effort, where the experimental design revealed specific causal factors for future study ([16], Table 1). As independent validation of genes from a single study can be very expensive and time consuming, reproducible expression across studies can also be used to identify reliable sex-biased genes. Accordingly, we set out to investigate whether there are robust sex-biased gene expression signatures in human brain by collecting and systematically integrating vast amount of publicly available data. Specifically, we collected transcription profiles for more than 16,000 samples from 46 datasets in human brain. By systematic integration of the data from multiple studies, we identified robust transcription level differences in human brain across 11 brain regions and classified male-biased and female-biased genes, and their likely origin and functional significance. We have also developed a web resource to make the entire analysis available for the scientific community for further exploration, available at https://joshiapps.cbu.uib.no/SRB_app/

## Methods

### Data collections and differential expression analysis

**Fig. 1:**
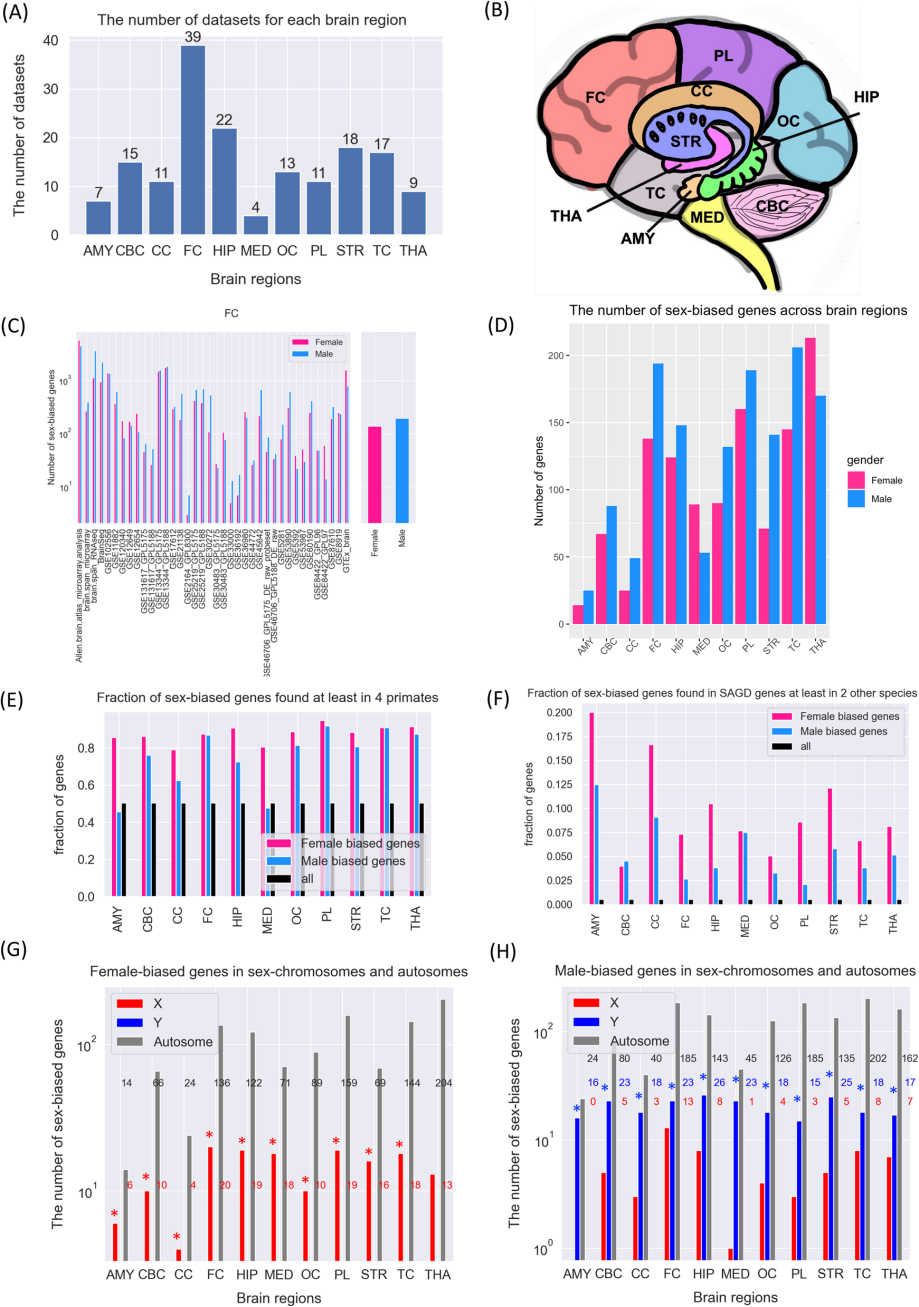
**A** The number of datasets analyzed in each brain region. **B** A schematic map of the brain regions studied. **C** The number of female-biased (pink) and male-biased (blue) genes for each dataset before and after rank aggregation. **D** The number sex-biased genes across brain regions. **E** Fraction of sex-biased genes found at least in 4 primates. **F** Fraction of sex-biased genes with sex-biased genes expression at least in 2 species. The number of genes on the sex chromosomes and autosomes in female-biased **G** and male-biased **H** genes

Gene expression datasets analyzed in this study were collected from several published brain studies (Fig. 1A and Additional file 1). The raw or normalised quantification matrix deposited alongside the original publication were re-processed and analyzed separately for all datasets. For the data obtained from the Gene Expression Omnibus (GEO) repository, the normalized gene expression were downloaded using the R package GEOquery 2.54.1 [17]. The microarray datasets with raw expression values were normalized and log transformed using Robust Multichip Average (RMA) method. Probes without a mapping gene were removed. The average expression value of gene with multiple probe sets was calculated. Differential expression analysis were performed separately by 11 brain regions: amygdala (AMY), cerebellum (CBC), frontal lobe (FC), hippocampus (HIP), medulla and spinal cord (MED), occipital lobe (OC), basal ganglia (STR), temporal lobe (TC), thalamus (THA), parietal lobe (PC), corpus callosum (CC). The empirical Bayes differential expression analysis was performed by using limma 3.42.0. A cutoff of fold-change at 1.2 and *p*-value of 0.05 were used to identify genes as significant female-biased genes and male-biased genes. Then, the female- and male-biased gene lists from each dataset were ranked by log fold-change from the rank aggregation method. The schematic diagram for methods in this study is shown in Additional file 4: Fig. S1. We used the sex annotation provided by available metadata for the samples in all datasets. We also performed principal component analysis (PCA) of sex-chromosome gene expression to confirm the accuracy of sex-labeling of samples. Additional file 4: Fig. S2 shows the example PCA plot of Y-chromosome genes for six datasets (GSE8397, GSE12649, GSE17612, GSE44456, GSE30483 and GSE45642). There were very rare instances of disagreement. The samples were omitted from the analysis in that case.

### Sex-biased gene prioritization by rank aggregation method

For each brain region, robust rank aggregation method (RRA) was used to combine multiple female- and male-biased rank gene lists from all datasets into a single prioritized female- and male-biased gene rank list [18]. For each gene, the status was assigned as a sex-biased gene using combined RRA rank selected by *p*-value less than 0.05.

We also applied another pipeline to define female- and male-biased genes. We firstly obtained differentially expressed genes by performing empirical Bayes differential expression analysis by using limma 3.42.0.A. All genes in each dataset were ranked based on fold-change to obtain male- and female-biased gene lists. The gene lists from each dataset were then combined using RRA. For each brain region, combined gene ranking from all gene ranks into one gene rank list using RRA and FDR corrected *P* value ${<}0.05$ from RRA used to filter sex-biased genes, i.e., female- and male-biased genes (Additional file 4: Fig. S27).

Correlations of sex-biased genes between brain regions were determined using Spearman’s correlation coefficient. Enrichment of sex-biased genes in X/Y-chromosomes and autosome were calculated using Fisher’s exact test. A conserved human gene list in six primates (’Bolivian squirrel monkey’, ’Chimpanzee’, ’Gorilla’, ’Gibbon’, ’Olive baboon’, ’Macaque’) from UCSC genome browser [19] was used to investigate the conservation of sex-biased genes. We used SAGD database [8] to check if sex-biased genes in human brain found in sex-biased genes of other species.

### Gene enrichment analysis and disease-related gene analysis

Gene Ontology (GO), Kyoto Encyclopedia of Genes and Genomes (KEGG) pathways significantly enriched in sex-biased genes were implemented using clusterprofiler (v.4.4.4) [20] at adjusted *p*-value smaller than 0.05 (corrected by the Benjamini–Hochberg method). Gene–disease association and disease enrichment of sex-biased genes were identified using DisGeNet2r package [21]. CURATED and all database options were used for disease enrichment analysis. The *p*-values resulting from the multiple Fisher’s exact tests are corrected for false discovery rate using the Benjamini–Hochberg method. The enrichment of Genome-Wide Association Studies (GWAS) Catalog 2019 were performed by enrichr [22]. We also performed over-enrichment analysis of sex-biased genes in a curated brain diseased and drug-target genes from BrainBase database [23] using Fisher’s exact test. To compare enriched terms across brain regions, top five significantly enriched categories of each brain regions were selected and plotted for visualizations for the enrichment. The gene count denoted by the size of the circle and adjust *p*-value denoted by the color. As enrichment analysis tools such as enrichr [22] do not allow user-defined background genes. We also tested whether we observed brain-specific functional categories enriched in DAVID online tool [24, 25] using all genes expressed in specific brain regions as a background.

### Multiple regression analysis with age and sex as independent variable

To study the contribution of age and sex only two datasets in frontal cortex had enough samples with a wide age range in both males and females. Therefore, only samples from frontal cortex brain region from GSE11882 [26] and GSE53890 datasets [27] were used for sex–age-related gene analysis. We model linear regression of each gene expression as linear combination of age, sex and (age*sex) variables as shown in Eq. 1:

$$\begin{aligned} \text{expression} = a * (\text{age}) + b * (\text{sex}) + c * (\text{age} * \text{sex}). \end{aligned}$$

Sex was created as a binary variable. Variable standardization was performed to reduce multicollinearity. Variance Inflation Factors (VIF) was used to test multicollinearity of the third independent variable with other independent variables. The cutoff of regression coefficient of age and sex variables were used to identify age- or sex-related genes (Additional file 4: Fig. S3).

### Cell-type and tissue-specific enrichment analysis

Enrichment of our sex-biased genes in two cell type-specific gene lists was calculated. First set of cell type-associated genes was from McKenzie et al. [28]. Fisher’s exact test was used to test for cell type-specific tissue. The second cell-type gene list was from Dougherty et al. [29]. In this section, specific expression analysis across cell types (CSEA) web tool was used to calculate Fisher’s exact test with Benjamini–Hochberg correction of the overlap between our sex-biased genes and their cell type-specific genes. In order to investigate whether sex-biased genes are highly enriched or specific expression in brain. Tissue-specific enrichment analysis (TSEA) was performed using deTS package with GTEx panel [30].

### Androgen response element (ARE), estrogen response element (ERE) and motif analysis

In order to determine the number of androgen (AR) and estrogen receptors (ER) in sex-biased genes, genes with full and half ARE and ERE binding sites from published studies [31, 32] were used to find an overlap genes between these receptor genes and sex-biased, brain expressed and brain regionally elevated genes (from the human protein atlas). Known motif enrichment analysis in the promoters of sex-biased genes was performed by HOMER (v4.11) [33].

### Drug–target interactions and adverse drug response

We used drug–target interactions with 2118 drugs/chemicals from BrainBase database [23]. Fisher’s exact test was used to calculate over-enrichment for drugs target in sex-biased genes. The enrich terms with *p*-value less than 0.0001 were plotted across all regions. The adverse drug reaction genes from Chen et al. [34] was also used to calculated overlap with sex-biased genes.

## Results

### Sex-biased gene expression across 11 human brain regions

Many studies have been published to identify sex-biased genes in one or several brain regions, but assessment of the robustness of these sex-biased genes is missing. In order to identify a robust sex-biased gene signature, i.e., sex-biased genes supported by multiple studies, we collected over 16,000 individual samples from 46 gene expression datasets in human brain (Fig. 1A). The samples were grouped into 11 brain regions namely, amygdala (AMY), cerebellum (CBC), frontal lobe (FC), hippocampus (HIP), medulla and spinal cord (MED), occipital lobe (OC), basal ganglia (STR), temporal lobe (TC), thalamus (THA), parietal lobe (PC), and corpus callosum (CC) (Fig. 1B). Individual datasets consisted diverse human sample material, experimental protocols and technology. We selected datasets with genome-wide expression data generated using either microarray or RNA sequencing technologies and with a minimum of ten samples of each sex. Technical and technological divergence across datasets, complicated pooling of samples. We therefore identified male-biased and female-biased genes in each individual dataset using differential expression analysis (*p*-value ${<}0.05$ and 1.2 or more fold-change) and further used robust rank aggregation method [18] to combine multiple ranked lists of sex-biased genes from different datasets (Additional file 4: Fig. S1), resulting into a robust male and female-biased gene list in each brain region. Fig. 1C (left box) shows the number of sex-biased genes (male—blue, female—pink) in each individual dataset, whereas Fig. 1C (right box) represents the number of sex-biased genes after the rank aggregation in the FC brain region (Additional file 4: Figs. S4 and S5 and Additional file 2 for female-biased genes and Addition file 3 for male-biased genes). We noted that there were only a handful of genes (${<}5$) detected as sex-biased across all studies in each brain region. This is likely due to heterogeneity of data caused by multiple factors. First and foremost, the heterogeneity across the human samples with diverse demographic and socioeconomic traits as well as the technological heterogeneity in the data including multiple platforms, different experiment protocols, and unequal sample size. Most of our sex-biased genes were identified as sex-biased in at least two datasets. There were on average about a hundred sex-biased genes in each of the 11 brain regions. THA, TC and FC with the most sex-biased genes while AMY and CC had the lowest number of sex-biased genes. There was no correlation between the number of sex-biased genes and the number of available datasets or the total number of samples across regions (Additional file 4: Fig. S6). Both male- and female-biased genes were present across all brain regions with a very small bias for male-biased genes than female-biased genes (Fig. 1D).

To estimate the conservation of sex-biased genes, we downloaded a list of conserved human genes in six primates (‘Bolivian squirrel monkey’, ‘Chimpanzee’, ‘Gorilla’, ‘Gibbon’, ‘Olive baboon’, ‘Macaque’) from UCSC genome browser [19]. About 80% of both male and female-biased genes were found in at least four primates compared to only about half of all human genes (black) conserved in at least four primates (Fig. 1E). Sex-biased genes in nearly all brain regions were therefore highly conserved across primates. We also estimated the conservation of sex-biased genes in higher eukaryotes from Ensembl. Lists of conserved human genes in 198 species were downloaded from Ensembl genome browser. Both male and female-biased genes were more conserved across other species than all genes (Additional file 4: Fig. S7). In order to check whether human sex-biased genes in brain show sex-biased expression in other species, we used SAGD database [8]. The SAGD database consists of sex-associated genes across organs in diverse species. Our human brain sex-biased genes were indeed enriched for sex-biased genes in other species. The fraction of male and female-biased with sex-associated genes in at least two species (excluding human) was significantly higher than all human genes (Fig. 1F).

We further checked whether sex-biased genes were enriched in specific genomic regions. Female-biased genes were significantly enriched on the X chromosome (Fig. 1G) and male-biased genes were enriched on the Y chromosome (Fig. 1H) as expected. For example, X chromosome contains about 5% of human genes and about 20% of female-biased genes in medulla were on the X chromosome. Similarly, Y chromosome contains about 1% of human genes and about 40% of male-biased genes in amygdala were on the Y chromosome. Furthermore, we checked whether the genes on sex chromosomes belonged to the pseudoautosomal regions (PAR1 and PAR2) of the human X and Y chromosomes which do recombine during meiosis. We noted that male-biased genes on Y chromosome and female-biased genes on X chromosome were not enriched for genes on pseudoautosomal regions (Additional file 4: Fig. S9A). XIST, a long non-coding RNA expressed from X chromosome ensures that one of the pair of X chromosomes is transcriptionally silenced (X chromosome inactivation or XCI) during early development in mammalian females. Many genes on X chromosome evade this dosage equivalence providing a mechanism for divergence between males and females, called XCI escape genes [35]. The female-biased genes on X chromosome highly overlapped with XCI escape genes (Additional file 4: Fig. S10B). In summary, we noted that sex-biased genes were enriched for sex chromosomes and were located on the sex-specific part of each chromosome. We noted no preference for autosomes for both male and female sex-biased genes.

**Fig. 2:**
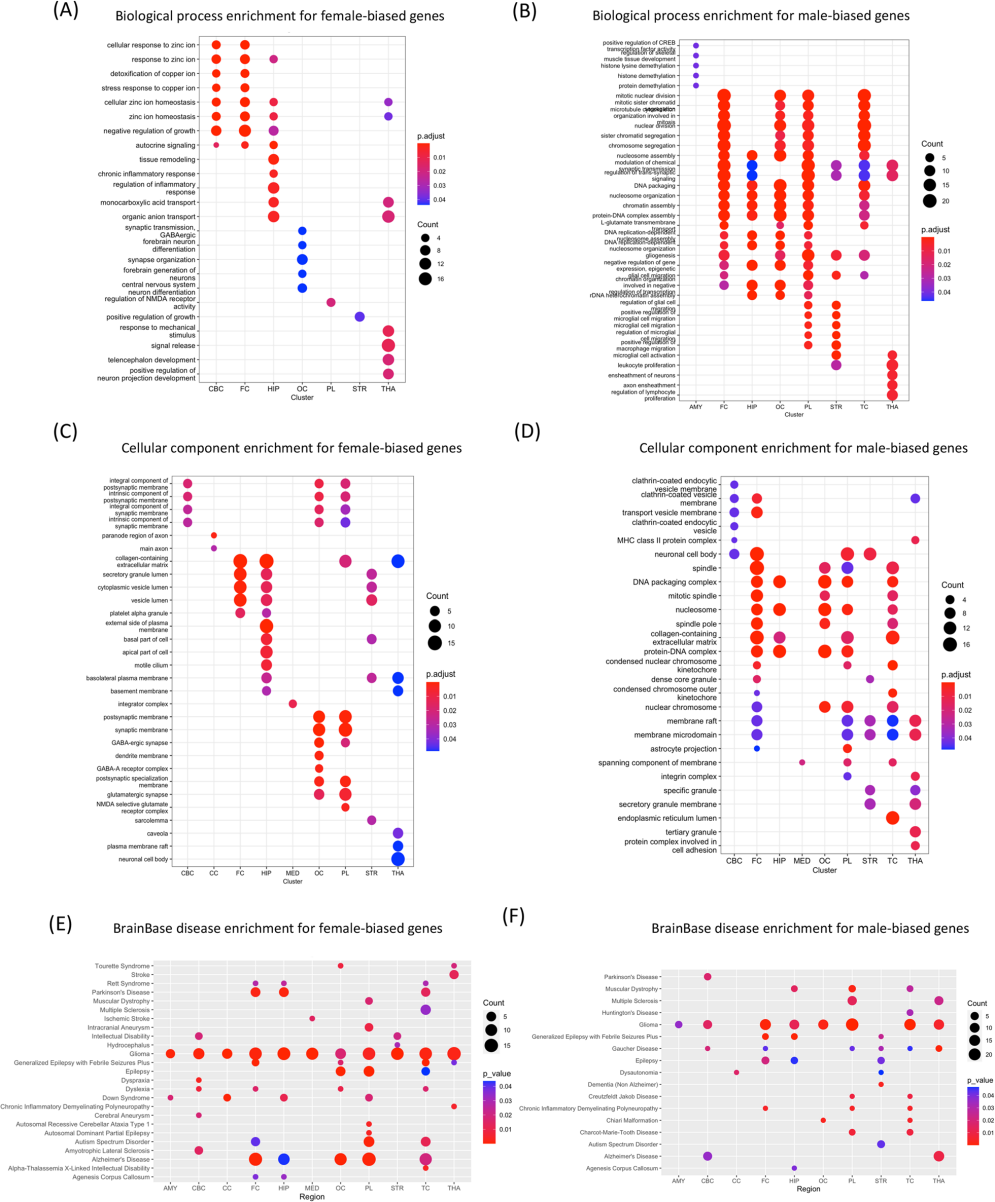
The top 5 enriched terms for the gene ontology and disease enrichment analysis across brain regions. **A** Biological process enrichment for female-biased genes. **B** Biological process enrichment for male-biased genes. **C** Cellular component enrichment for female-biased genes **D** Cellular component enrichment for male-biased genes. **E** BrainBase disease enrichment analysis for female-biased genes. **F** BrainBase disease enrichment analysis for male-biased genes

To explore the functional relevance of sex-biased genes, we first conducted the pathway enrichment analysis using Gene Ontology (GO) and Kyoto Encyclopedia of Genes and Genomes pathway annotations (Fig. 2, bigger figure shown in Additional file 4: Figs. S11–S14). The functional enrichment of female-biased genes was brain region specific with CBC and FC genes enriched for zinc and copper response while OC genes for neuronal activity (Fig. 2A, B and Additional file 4: Fig. S11). In cellular component enrichment analysis, the most significant enrichment terms of female-biased genes were related to postsynaptic and synaptic membrane in OC and PL and lumen in FC and HIP (Fig. 2C and Additional file 4: Fig. S12A). While male-biased genes were related to DNA packaging, spindle and nucleosome in many brain regions (Fig. 2D and Additional file 4: Fig. S12B). In summary, female-biased genes were enriched for neuron-associated processes while male-biased genes were enriched for nuclear structures. Enrichment of Disease-Related Genes in sex-biased genes across brain regions were examined using BrainBase, DisGeNet (curated) and GWAS catalog 2019 database (Fig. 2E, F, and Additional file 4: Figs. S15, 16). Sex-biased genes were highly enriched for genes related to glioma across many brain regions (Fig. 2E, F). Alzheimer’s related genes were highly enriched for the female-biased genes across four brain regions (Fig. 2E). SFARI database (https://gene.sfari.org/) [36] contains about 1000 genes related to autism spectrum disorders (ASD). Female-biased genes in several brain regions were enriched for ASD-related genes (Additional file 4: Fig. S17).

### Sex-biased gene overlap across brain regions

**Fig. 3:**
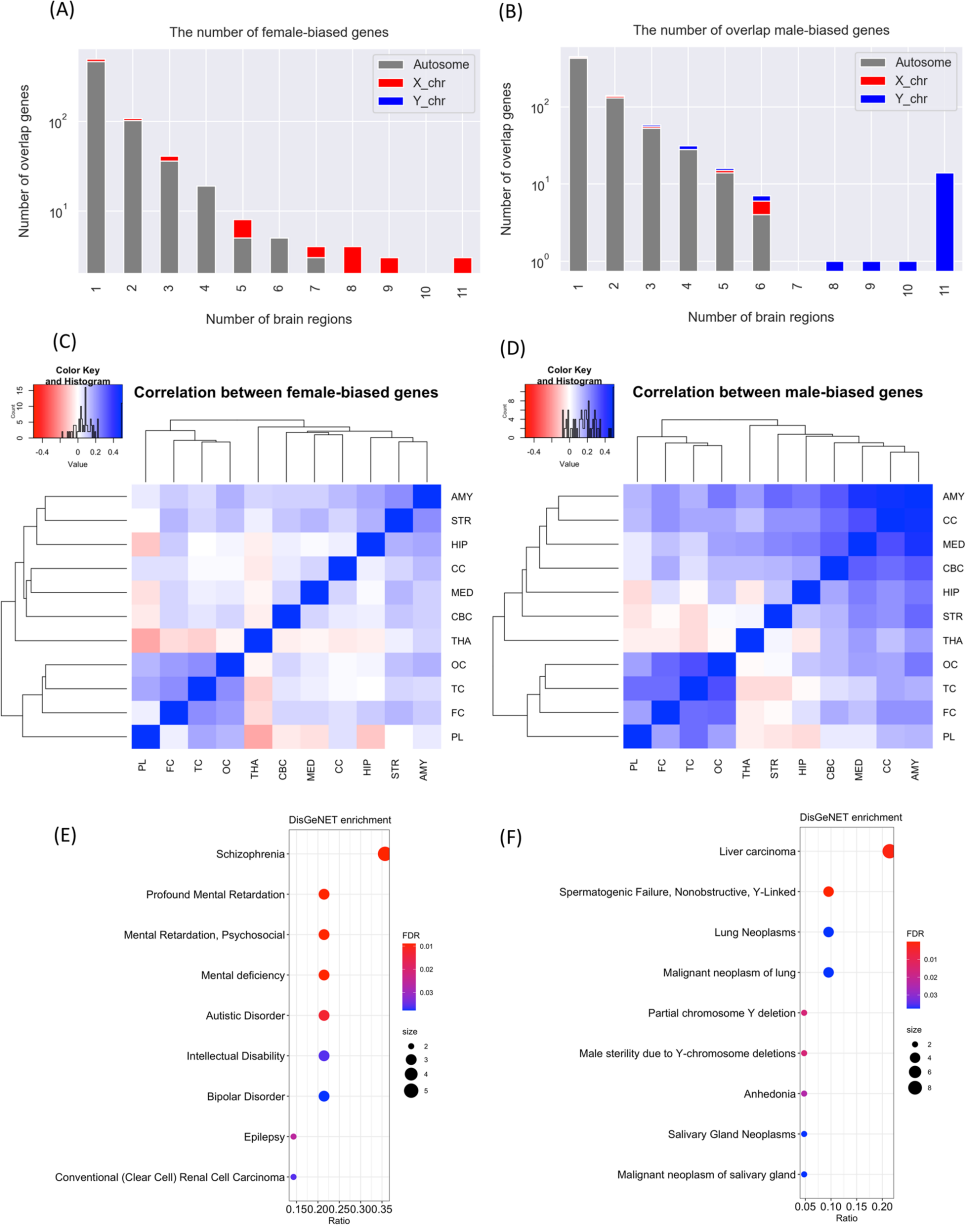
**A** The number of female-biased genes by number of regions. The color grey, red and blue are shown bar graphs for proportions of genes mapped into autosome, X-chromosome and Y-chromosome, respectively. **B** The number of male-biased genes by number of regions. **C** The correlation heatmap of female-biased genes. **D** The correlation heatmap of male-biased genes. **E** DisGeNet (CURATED) enrichment of overlap female-biased genes across FC, PL, TC and OC **F** DisGeNet (CURATED) enrichment for overlap female-biased genes across FC, PL, TC and OC

Given that functional enrichment showed high overlap across male-biased genes, we hypothesized high overlap among male-biased gene sets compared to female-biased gene sets across brain regions. Indeed, the overlap of sex-biased genes showed that male-biased genes were more shared with 14 genes were male-biased in all 11 brain regions (Fig. 3B) compared to only three genes were female-biased in all brain regions (Fig. 3A). Importantly, female- and male-biased genes found in more than eight brain regions were located on X- and Y-chromosomes, respectively.

We further calculated a pair-wise overlap of male and female-biased genes across brain regions. Female-biased genes in each brain region showed very little overlap with other brain regions (Fig. 3C), while the male-biased genes grouped the brain regions in two core clusters (Fig. 3D). Male-biased genes in AMY, CC and MED showed a high overlap. We noted that sex-biased genes in PL, FC, TC and OC showed a distinct signature in both males and females with a high correlation between these brain regions (Fig. 3C, D). Overlap sex-biased gene lists of PL, FC, TC and OC were further examined for gene-disease association enrichment from databases GWAS catalog 2019 and DisGeNet (curated database) (Fig. 3E, F and Additional file 4: Fig. S18). The female-biased genes in four regions were enriched for Alzheimer’s disease progression (SYN3 and STK32B) and the male-biased genes in four brain regions were enriched for neuroticism (PAX6 and PLTP). The DisGeNet enrichment for female-biased genes in four brain regions identified many mental disorders (Fig. 3E, Additional file 4: Fig. S19A).

### Cell-type and tissue specificity of sex-biased genes

So far, we identified robust sex-biased genes and noted that male-biased genes across brain regions showed higher overlap in the previous sections. To check whether sex-biased genes show brain-specific gene expression, we performed tissue enrichment analysis using deTS [30]. The overlap of tissue-specific genes and sex-biased genes revealed that both male and female-biased genes significantly overlapped with brain-specific genes. Specifically, in almost all brain regions, brain tissues were the only enriched tissues out of a total of 48 body tissues for sex-biased genes (Additional file 4: Fig. S20). However, the sex-biased genes were not enriched for the genes specific to the individual brain regions, i.e., sex-biased genes in hippocampus did not show highest enrichment for deTS hippocampus genes. In summary, we observed that the sex-biased genes were brain specific compared to other body tissues but not brain region specific within the brain (Additional file 4: Fig. S20).

**Fig. 4:**
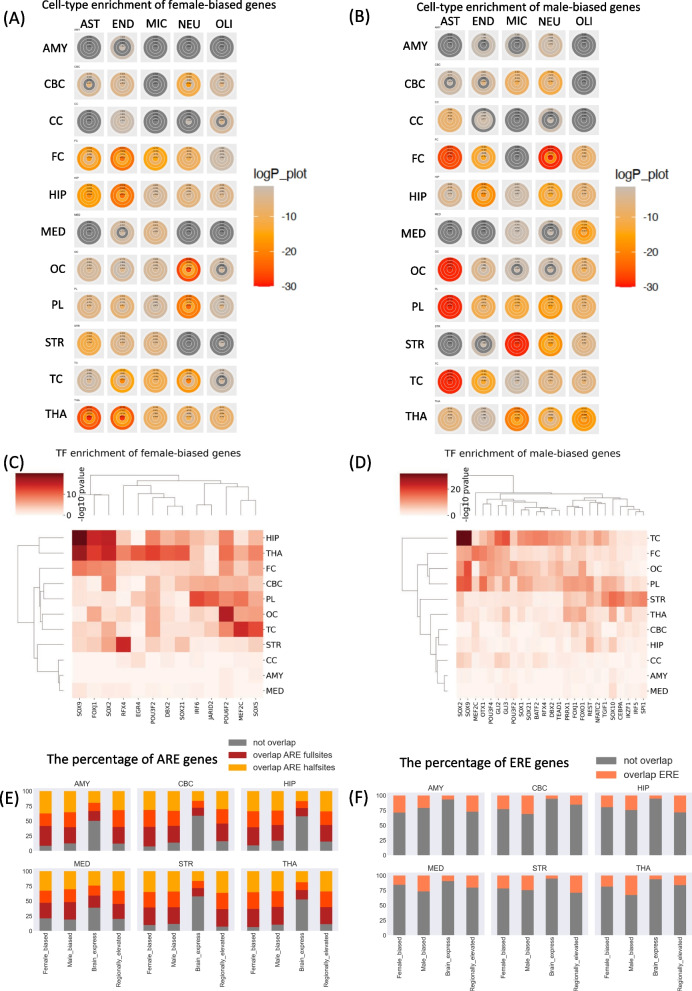
**A** Cell-type enrichment for female-biased genes. **B** Cell-type enrichment for male-biased genes. **C** Transcription factor (TF) enrichment for female-biased genes. **D** Transcription factor (TF) for male-biased genes. **E** The percentage of overlap between androgen receptor element (ARE) genes and sex-biased genes, brain expressed genes, and brain regionally elevated genes. The colors grey, red, yellow and orange in bar graphs represent the proportion of genes that not overlap, overlap with ARE full sites genes, overlap with ARE half sites genes and overlap both in ARE full and half sites, respectively. **F** The percentage of overlap between estrogen receptor element (ERE) genes and sex-biased genes, brain expressed genes and brain regionally elevated genes. The color of grey and peach are shown the proportion of genes that not overlap and overlap with ERE genes, respectively

Given that sex-biased genes were enriched for brain-specific genes, we further explored whether there was a cell-type specificity for sex-biased genes in brain. We used brain cell signature gene lists for five cell types (astrocytes, oligodendrocytes, microglia, neurons and endothelial cells) from McKenzie et al. gene sets [28] and calculated significant overlap using different thresholds for both male and female-biased genes (see "Methods"). As expected, both male and female-biased genes were enriched for many cell-type signature genes across brain regions (Fig. 4A, B bigger figure shown in Additional file 4: Fig. S21)). We noted that male-biased genes across brain regions were enriched for astrocytes and oligodendrocyte signature genes more than female-biased genes. A previous study exploring genes exhibiting sex-biased expression in human fetal brain, noted that the male-biased genes were enriched for expression in neural progenitor cells, whereas female-biased genes are enriched for expression in Cajal–Retzius cells and glia [37]. This observation was not supported in our analysis of adult brain regions. We also used an independent resource of cell type-specific expression in human brain [38] to calculate cell type enrichment of male and female-biased genes (Additional file 4: Fig. S22–S25). Indeed, male-biased gene enrichment for astrocytes and oligodendrocytes in FC, OC, PL and TC is supported by both the datasets (Fig. 4B).

### Regulatory mechanisms behind sex-biased gene expression

**Fig. 5:**
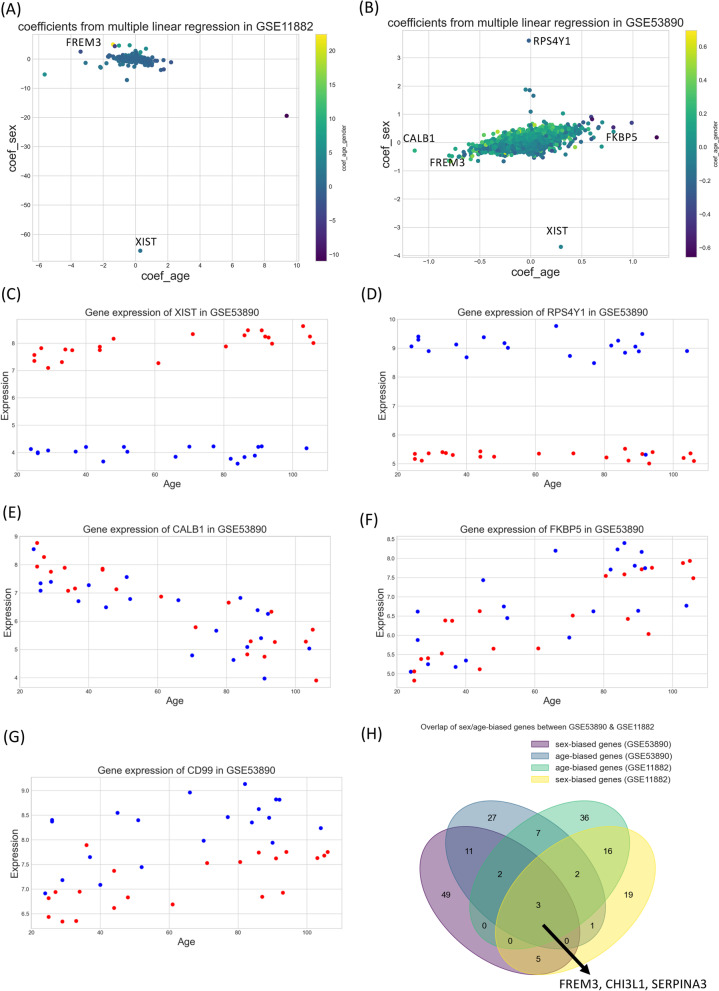
**A** The scatter plot of the coefficients of age and gender variables from the multiple linear regression from GSE11882. **B** The scatter plot of the coefficients of age and gender variables from the multiple linear regression from GSE53890 dataset. **C** Gene expression of XIST gene in GSE53890 dataset, labeled as red and blue for female and male samples, respectively. **D** Gene expression of RPS4Y1 genes in GSE53890 dataset, colored red and blue for female and male samples, respectively. **E** Gene expression of CALB1 genes in GSE53890 dataset, colored red and blue for female and male samples, respectively. **F** Gene expression of FKBP5 genes in GSE53890 dataset, colored red and blue for female and male samples, respectively. **G** Gene expression of FKBP5 genes in GSE53890 dataset, colored red and blue for female and male samples, respectively. **H** Venn diagram of overlap genes between sex-biased genes and age-biased genes from GSE53890 and GSE11882 datasets

To explore possible transcription regulatory mechanisms behind sex-biased genes, we firstly performed known motif enrichment analysis in the promoters of the sex-biased genes. The analysis did not identify strong enrichment for motifs of specific transcription factors (Additional file 4: Fig. S18) for both male and female-biased gene sets. We then obtained a reconstructed transcription regulatory network model in human brain by integrating brain-specific DNase footprinting and TF-gene co-expression [39]. This network consisted of over 700 transcription factor and their predicted targets. The enrichment analysis of predicted transcription factor targets in male and female-biased gene list identified many potential transcription factors (Fig. 4C, D). Male-biased genes in TC, FC, OC and PL had a high overlap. Accordingly, many transcription factors, notably SOX family member targets were enriched in these four regions in male-biased genes (Fig. 4D). SOX2 and SOX9 putative targets highly overlapped with female-biased genes in two brain regions (HIP and THA) (Fig. 5C). Interestingly, female-biased genes in HIP and THA had nearly no overlap (Fig. 3C).

Sex-specific hormones can mediate sex-biased gene expression. We therefore obtained genes enriched for the hormone response elements. The overlap between our robust sex-biased genes and androgen response elements (ARE) and estrogen response elements (ERE) was calculated. We noted that over 80% of both male and female-biased genes were enriched for either half or full ARE sites across brain regions (Fig. 4E). This fraction is significantly higher than all human genes with about 50% genes with ARE half or full sites (Fig. 4E). We also obtained gene lists with highly expressed genes in specific brain regions called regionally elevated genes from Allan Brain Atlas. This genes showed similar enrichment to sex-biased genes for ARE half and full sites (Fig. 4E). The analysis of ERE binding sites provided with results similar to ARE binding sites, i.e., sex-biased and regionally elevated genes were enriched for ERE sites compared to all genes (Fig. 4F). In summary, sex-biased genes are enriched for sex hormone response elements.

### Age and sex relationship in brain gene expression

We previously noted that one of the likely reasons for the low overlap in sex-biased genes across different studies is the fact that brain samples came from very diverse human cohorts with heterogeneity in many socio-demographic traits including age. To dissect, sex and age components, we selected datasets covering samples in a wide age range for both sexes. Only two datasets from the human frontal cortex provided sample variability in age to allow estimation of age and sex effect on the gene expression. We therefore evaluated the effect the sex and age on brain gene expression using two datasets; GSE11882 [26] and GSE53890 [27]. Multiple linear regression for individual genes was performed using age, sex and age*sex as independent terms in each dataset (Methods for details). The coefficients for the age and sex terms were used to select sex-biased and age-biased genes (Fig. 4A, B). The regression coefficient for age*sex term for about 50% genes was greater than individual age or sex variable, demonstrating that both age and sex influence gene expression for most genes.

We noted that XIST was highly sex specific and female biased (Fig. 5C) and genes on Y chromosome—ribosomal protein S4 (RPS4Y1), KDM5D, USP9Y and DDX3Y were male-biased, as expected (Fig. 5D). On the other hand, some genes showed expression variability mainly through aging. For example, calcium binding protein, CALB1 decreased during aging (Fig. 5E), while immune regulatory gene FKBP5 increased during aging (Fig. 5F) consistently in both males and females. We noted that many female-biased genes decreased gene expression during aging while many male-biased genes increased in gene expression. For example, Cluster of differentiation 99 (CD99) expression was male-biased and increased during aging (Fig. 5G). We identified age and sex-associated genes in each dataset (see Methods) and calculated overlap between them (Fig. 5H). Only 8 genes were sex associated in both datasets (FREM3, DDX3Y, KDM5D, SERPINA, USP9Y, XIST, CHI3L1, EIF1AY) and fourteen genes were age associated in both datasets (CBLN4, FREM3, AKAP5, C11orf87, CRH, LINC00507, SERPINA3, CALB1, RGS4, CHI3L1, AQP1, VIP, S100A8, NETO2). Age and sex-associated genes had a high overlap in each dataset (Fig. 5H) and three genes were found age- and sex- associated in both datasets. FREM3 was female-biased while CHI3L1 and SERPINA3 genes were male-biased, and the expression of CHI3L1 and SERPINA3 increased with age while the expression of FREM3 genes decreased with age. All these genes have been shown to be associated with neurological disease [40–42]. FREM3 is associated with depression and aging in human brain [40]. Another study also found sex-, age- and Alzheimer’s disease-related differences in CHI3L1 expression in the brain. Interestingly, CHI3L1 is highly expressed in female AD patients compared to male AD samples [41].

### Sex-biased drug response

**Fig. 6:**
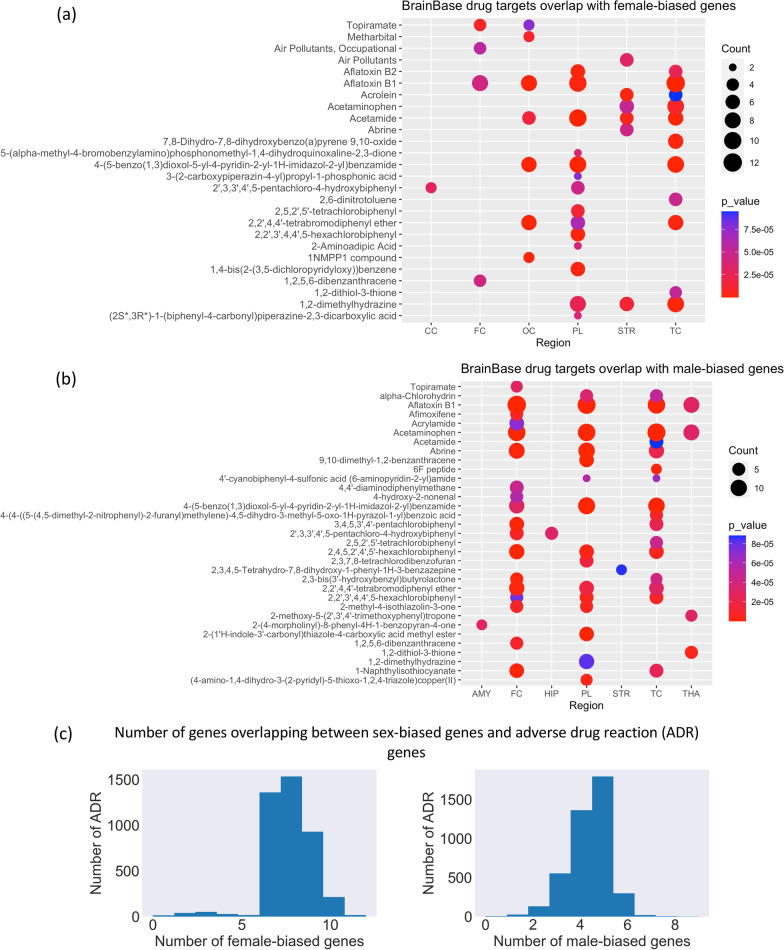
**A** Over-enrichment of BrainBase drug targets for female-biased genes. **B** Over-enrichment of BrainBase drug targets for male-biased genes. **C** Number of genes overlapping between female-biased genes and adverse drug reaction genes (left). Number of genes overlapping between male-biased genes and adverse drug reaction genes (right)

After evaluating the likely regulatory factors of sex-biased genes, we explored the clinical impact of sex-biased expression. It is well documented that males and females have differential response to many drugs. We used drug–target interactions covering 2118 drugs or chemicals and 623 genes from BrainBase database [23] to calculate the enrichment for drugs in sex-biased genes. Many drug targets were enriched for sex-biased genes (*P* value ${<}0.0001$) in both males and females, particularly in FC, PL and TC brain regions. More female-biased genes overlapped with drug targets than male-biased genes. For example, midazolam target genes were female-biased in FC and aspirin targets were male-biased in TH. Indeed, midazolam, a sedative and anesthetic adjuvant, has demonstrated sex-specific effects with deeper sedation in men compared with women [43] and sex difference in aspirin response is also well known where women are 2.5 times more likely to be aspirin resistant than men [44]. Cisplatin targets were enriched in male-biased genes in temporal and occipital CC. Cisplatin-related gender differences in nephrotoxicity also showed greater damage in males than females [45]. Antipsychotic and antidepressant targets were enriched for female-biased genes. There are known sex differences in pharmacodynamic effects of many drugs. In women, they include greater sensitivity to and enhanced effectiveness of beta blockers, opioids, selective serotonin reuptake inhibitors, and typical antipsychotics. Additionally, women are 50–75% more likely than men to experience an adverse drug reaction [46]. We therefore further explored whether the genes associated with adverse drug reactions were sex-biased. We obtained adverse drug reaction genes from Chen et al. [34] and calculated overlap with sex-biased genes. Both male and female-biased genes overlapped with many adverse drug reaction phenotypes (Fig. 6C, D, y axis). However, female-biased genes showed a higher overlap of genes for most of adverse drug reaction phenotype (Fig. 6C).

### A web resource of sex-biased gene expression analysis in human brain

We developed a publicly available web resource to provide access to the key analysis of sex-biased genes. SexRankBrain is an R shiny interactive tool [47] to explore the sex-biased genes across datasets from human brain. This web resource, in addition also allows the robustness analysis of our findings as it allows users to change different thresholds during the analysis. We utilized this feature of the web resource to confirm that major finding noted in this study were consistent at different thresholds. Users can set thresholds to obtain sex-biased gene lists from all datasets for each brain regions in this study. These lists can then be used in the web application for calculating sex-biased gene rank using custom parameter from user and create a result dashboard. There are three module tabs in the application. The first and second tab allow users to explore the functional features of sex-biased genes for individual brain regions as well as a comparison of sex-biased genes across all brain regions, respectively. The third tab contains information about web-application. In the first tab, the web-app allows user to select their preference cutoff for a specific brain region in three steps. First step is to apply *p*-value and logFC cutoff for sex-biased genes filtering in all datasets. Next, the web application performs a robust rank aggregation (RRA) from all gene ranks and creates a combined sex-biased gene rank [18] for each brain region. Users can choose a custom RRA p-value cutoff to filter significant sex-biased genes from an aggregate rank. The last step is to perform diverse enrichment analyses of significant sex-biased genes. Gene ontology (GO), Kyoto Encyclopedia of Genes and Genomes (KEGG) pathways and DisGeNet [21] enriched in sex-biased genes are implemented using Enrichr [22]. The web application has a second tab for the comparison of sex-biased genes across brain regions. Here, users can similarly choose parameters for the steps as described in the first tab. The enrichment results for this tab are implemented by compareCluster() function in clusterProfiler package [20]. For both tabs, the tables of custom filtered rank genes and all individual figures can be downloaded. The open source code for the shiny application is available on GitHub (https://github.com/PattaWapee/SexRankBrain).

## Discussion

Males and females display a plethora of divergent physical and behavioral patterns, affecting many life outcomes including disease prevalence, symptoms, and progression rates. Funding agencies and publishers are calling for greater attention to exploring basic biological processes and disease mechanisms across the sexes and genders. Besides the reproductive organs, most sex differences in the body are quantitative, i.e., the distribution differ between the two sexes but largely overlaps, as is the case with the height or the brain volume, as well as many other physiological traits such as stress, opioid sensitivity, and immune response [16]. Accordingly, studies exploring sex differences during development and disease in humans have exploded in recent years. Nevertheless, most of them derived their conclusions based on only single or a handful of datasets [7–10]. A recent study [10] noted sex differences across many cell types in humans including brain, albeit only using one dataset, unlike this study. The study [10] did not focus specifically on the brain but many of our findings were indeed overlapping, e.g., enrichment of sex-biased genes on sex chromosomes. The sex differences in brain are controversial mainly because of its likely societal implications. Women have been culturally oppressed and sex differences have been used as a justification for some of the injustices. Nevertheless, the sex differences in the brain may explain the differences seen in prevalence, symptomology and even treatment for brain pathologies. It is therefore important to establish the validity of observed sex differences across different studies. We therefore combined a huge amount publicly available expression data to estimate sex differences in human brain. Firstly and most importantly, we noted that most of sex-biased genes obtained from one data were not identified in other datasets. This is partially due to heterogeneity in data, e.g., the impact of the age of individuals (explored in detail in this paper), as well as other confounders, and technical and technological differences across studies. By systematic data integration, we obtained a robust sex-biased gene list for each of 11 brain regions. The robust sex-biased genes were highly conserved and showed sex-biased gene expression in other species as well. This allowed us to validate some of the findings obtained previously using individual datasets as well as generated some novel hypotheses. We first established that our findings are not sensitive to a specific threshold of the analysis pipeline (analysis available as a web resource). Furthermore, we used another independent pipeline to define sex-biased genes (see Methods for details and Additional file 4: Fig. S27). Using this alternative pipeline (Additional file 4: Fig. S27), we validated that main findings described in this manuscript are not dependent on the specifics of the analysis pipeline (Additional file 4: Figs. S27–30), providing additional confidence in the findings.

Arnold proposed a general theory of mammalian sexual differentiation whereby sex chromosome genes are the primary factors causing sexual differentiation [3]. The biggest genetic distinguishing factor between two sexes is the presence of sex chromosome where x chromosome causes sex differences in gene expression through XIST, X genes escaping inactivation, and imprinted X genes [3]. For example, Kassam et al. [9] observed that X-linked KAL1 gene had higher expression in females than males in lung tissue. The biallelic expression of KAL1 gene in lung tissue is an example of tissue-specific escape from X-activation [48]. Indeed, the sex-biased genes in females were enriched on the X chromosome and particularly for the XCI escapee genes. This suggests that a part of sex-biased gene expression originates from the XCI escape mechanisms. Also, male-biased genes in brain were enriched for Y chromosome. On the other hand, it was noted that 90% of sex-biased genes across human tissues were mapped to autosomes, thus it’s not restricted within sex chromosomes [15]. This finding was partly supported in our study where most sex-biased genes in all brain regions were expressed from autosomes, rather than sex chromosomes. It is important to note that our robust sex-biased genes contained a higher fraction of genes (15–40%) on sex chromosomes, i.e., genes on sex chromosomes are more likely to be validated across studies and across multiple tissues. Furthermore, sex-biased genes found in more than eight brain regions were primarily on the sex chromosomes. X chromosome is particularly enriched for genes involved in brain-related functions. Many functional enrichment analysis tool including enrichr do not allow user-defined background genes. We therefore validated the brain-related functional enrichment in the sex-biased genes using DAVID online tool [24, 25] by providing brain region specific background genes (Additional file 4: Fig. S31).

Sex chromosomes are thought to regulate gene expression manifesting sex differences in brain primarily through the steroid hormones. Accordingly, we noted that both male and female-biased genes were enriched for both androgen and estrogen receptor binding sites. These findings departs from the traditional model of testosterone masculinizes the brain of males away from a default female form and supports a model where sex effects on the brain of both females and males are exerted by genetic, hormonal, and environmental factors. These factors act via multiple partly independent mechanisms that may vary according to internal and external factors [49].

We further tested the specific cell type enrichment for the robust sex-biased genes. Our results are in somewhat agreement with the previous reports that non-neuronal cells and inflammatory mediators were found in greater number and at higher levels in male brains [50]. The higher baseline of inflammation is speculated to increase male vulnerability to developmental neuropsychiatric disorders that are triggered by inflammation [50]. We noted a strong male bias for astrocytes and oligodendrocytes but not microglia. Nevertheless it is important to note that, gene expression is affected many factors. Kang et al. [51] studied the spatio-temporal dynamics of the human brain transcriptome to note that age contributed more to the global differences in gene expression than sex. For example, in middle-aged women, the gene expression changes were higher for astrocytes, endotheliocytes, and microglia compared to young women [52]. We performed a systematic analysis of the two datasets to estimate the effect of age and sex on the gene expression to note that for most genes sex and age both influence expression. Some age-related traits are conserved across sexes, there is age-related activation of immune- and inflammation-related genes in both male and female brains [26], while others are affected by both sex and age. Males showed significantly more gene expression changes in brain through aging with substantial gene change in the transition to the sixth and seventh decades of life. In contrast, females showed the largest numbers of genes responding in the eighth and ninth decades of life [26]. Schizophrenia has a more severe course (negative symptoms as well as cognitive impairment), experienced earlier in life in boys than in girls [53]. We explored the clinical implications the sex differences and noted that female-biased genes showed a high overlap with Alzheimer-related genes. Importantly, we also noted that more female-biased genes are involved in adverse drug reactions. Despite funders (e.g. NIH) pushing for female inclusion in clinical studies, very few (less than 10 percent of studies) are examining health issues related to females [54].

## Perspectives and significance

In summary, by integrating large amount of expression data, we identified robust sex differences across human brain regions. We have made entire analysis available as a web resource at https://joshiapps.cbu.uib.no/SRB_app/ for further exploration and hypothesis generation. Sex, together with age and other factors, affects brain function through human life span. Heterogeneity of human samples in many gene expression cohorts therefore makes it challenging to discern exactly the sex component. This indeed is a major shortcoming of many studies including this one. The finding of this study emphasized the importance of the need for greater attention to exploring basic biological processes and disease mechanisms in a sex and gender context. This study provided a foundation for future research to further investigate the mechanisms and factors contributing to sex and gender differences in the human brain.

## Supplementary Information

**Additional file 1.** List of published datasets used in this study.

**Additional file 2.** The table of female-biased gene in each brain regions with chromosome information and check-tick in all datasets used in this study.

**Additional file 3.** The table of male-biased gene in each brain regions with chromosome information and check-tick in all datasets used in this study.

**Additional file 4: Figure S1.** Schematic diagram of the workflow including rank aggregation for sex-biased genes detection in this study. **Figure S2.** Principal component analysisplot of Y- chromosome genes expression for GSE8397, GSE12649, GSE17612, GSE44456, GSE30483 and GSE45642. **Figure S3.** The cutoff for coefficient of age and sex variables in section multiple regression analysis with age and sex as independent variable. **Figure S4.** The number of sex-biased genes in individual datasets, aggregated lists in AMY, CBC, CC, HIP, MED and OC. **Figure S5.** The number of sex-biased genes in individual datasets, aggregated lists in PL, STR, TC and THA. **Figure S6.** Plot of number of sex-biased genes in different number of brain samples. **Figure S7.** Fraction of sex-biased genes found at least in 40 species and 160 species. **Figure S8.** The number of overlapping sex-biased genes by number of datasets for AMY, CBC, CC, FC, HIP and MED. **Figure S9.** The number of overlapping sex-biased genes by number of datasets for OC, PL, STR, TC and THA. **Figure S10.** The number of par1 genes in male-biased genes and the number of XCI female-biased genes. **Figure S11.** Gene ontology enrichment analysis of sex-biased genes for biological process. The top 5 enrich terms across brain regions in female-biased genes and male-biased genes. **Figure S12.** Gene ontology enrichment analysis of sex-biased genes for cellular component. The top 5 enrich terms across brain regions in female-biased genes and male-biased genes. **Figure S13.** Gene ontology enrichment analysis of sex-biased genes for molecular functions. The top 5 enrich GO terms across brain regions in female-biased genes and male-biased genes. **Figure S14.** KEGG pathway enrichment analysis of sex-biased genes. The top 5 enrich GO terms across brain regions in female-biased genes and male-biased genes.**Figure S15.** GWAS catalog 2019 enrichment across brain regions in female-biased genes and male-biased genes. **Figure S16.** DisGeNET enrichment analysis of sex-biased genes across brain regions in female-biased genes and male-biased genes. **Figure S17.** The number of autism genes found in sex-biased genes. Autism genes come from SFARI database. **Figure S18.** DisGeNetenrichment of overlap sex-biased genes across FC,PL,TC and OC in female-biased genes and male-biasd genes. GWAS catalog 2019 enrichment analysis of overlap sex-biased genes across FC, PL, TC and OC in female-biased genes and male-biased genes. **Figure S19.** Gene-disease class heatmap of DisGeNET for overlap sex-biased gene lists of PL, FC, TC and OC. Diseases are grouped by the their MeSH disease classes. The color scale is related to the percentage of disease in each class. **Figure S20.** Tissue-specific enrichment analysisof female-biased genes and male-biased genes. **Figure S21.** Cell type enrichment of sex-biased genes from McKenzie et al gene sets. **Figure S22.** Cell type specific expression analysisof female biased genes in AMY, CBC, CC, FC, HIP and PL. **Figure S23.** Cell type specific expression analysisof female biased genes in STR, TC and THA. **Figure S24.** Cell type specific expression analysisof male biased genes in CBC, CC, FC, HIP, MED and OC. **Figure S25.** Cell type specific expression analysisof male biased genes in PL, STR, TC and THA. **Figure S26.** Known motif enrichment analysis by HOMER in the promoters of the female-biased genes and male-biased genes. **Figure S27.**Schematic diagram of additional workflow to generate sex-biased gene list.The number of sex-biased genes from the additional workflow. **Figure S28.**The number of female-biased genes across brain regions using additional workflowThe number of male-biased genes across brain regions using additional workflow.The correlation heatmap of female-biased genes using additional workflow.The correlation heatmap of male-biased genes using additional workflow.The percentage of overlap between Androgen receptor elementgenes and sex-biased genes, brain expressed genes, and brain regionally elevated genes using additional workflow.The percentage of overlap between Estrogen receptor elementgenes and sex-biased genes. brain expressed genes and brain regionally elevated genes using additional workflow. **Figure S29.**Fraction of sex-biased genes found at least in 40 primates using additional workflow.Fraction of sex-biased genes found at least in 160 primates using additional workflow.Gene ontology enrichment analysis of female-biased genes for biological process using additional workflow.Gene ontology enrichment analysis of male-biased genes for biological process using additional workflow.Gene ontology enrichment analysis of female-biased genes for molecular function using additional workflow.Gene ontology enrichment analysis of male- biased genes for molecular function using additional workflow. **Figure S30.**Gene ontology enrichment analysis of female-biased genes for cellular composition using additional workflow.Gene ontology enrichment analysis of male-biased genes for molecular cellular composition using additional workflow.DisGeNetenrichment of female-biased genes using additional workflow.) DisGeNetenrichment of male- biased genes using additional workflow. **Figure S31.** Gene ontology analysis of output from DAVIDfor biological process.The top GO terms in female-biased genes in THA region with background genes of THA gene expression data.The top GO terms in male- biased genes in THA region with background genes of THA gene expression data.

## Data Availability

The raw expression data are published and we have made the analysis for this study available at https://joshiapps.cbu.uib.no/SRB_app/
