# Supplementary material for: Integrated analysis of robust sex-biased gene signatures in human brain
Source: Biol Sex Differ. 2023 May 24;14:36. doi: 10.1186/s13293-023-00515-w (PMC10207743; doi:10.1186/s13293-023-00515-w)
Supplement: Supplementary file 4 — Additional file 4: Figure S1. Schematic diagram of the workflow including rank aggregation for sex-biased genes detection in this study. Figure S2. Principal component analysisplot of Y- chromosome genes expression for GSE8397, GSE12649, GSE17612, GSE44456, GSE30483 and GSE45642. Figure S3. The cutoff for coefficient of age and sex variables in section multiple regression analysis with age and sex as independent variable. Figure S4. The number of sex-biased genes in individual datasets, aggregated lists in AMY, CBC, CC, HIP, MED and OC. Figure S5. The number of sex-biased genes in individual datasets, aggregated lists in PL, STR, TC and THA. Figure S6. Plot of number of sex-biased genes in different number of brain samples. Figure S7. Fraction of sex-biased genes found at least in 40 species and 160 species. Figure S8. The number of overlapping sex-biased genes by number of datasets for AMY, CBC, CC, FC, HIP and MED. Figure S9. The number of overlapping sex-biased genes by number of datasets for OC, PL, STR, TC and THA. Figure S10. The number of par1 genes in male-biased genes and the number of XCI female-biased genes. Figure S11. Gene ontology enrichment analysis of sex-biased genes for biological process. The top 5 enrich terms across brain regions in female-biased genes and male-biased genes. Figure S12. Gene ontology enrichment analysis of sex-biased genes for cellular component. The top 5 enrich terms across brain regions in female-biased genes and male-biased genes. Figure S13. Gene ontology enrichment analysis of sex-biased genes for molecular functions. The top 5 enrich GO terms across brain regions in female-biased genes and male-biased genes. Figure S14. KEGG pathway enrichment analysis of sex-biased genes. The top 5 enrich GO terms across brain regions in female-biased genes and male-biased genes.Figure S15. GWAS catalog 2019 enrichment across brain regions in female-biased genes and male-biased genes. Figure S16. DisGeNET enrichment analysis of sex-biase [file 13293_2023_515_MOESM4_ESM.pdf]

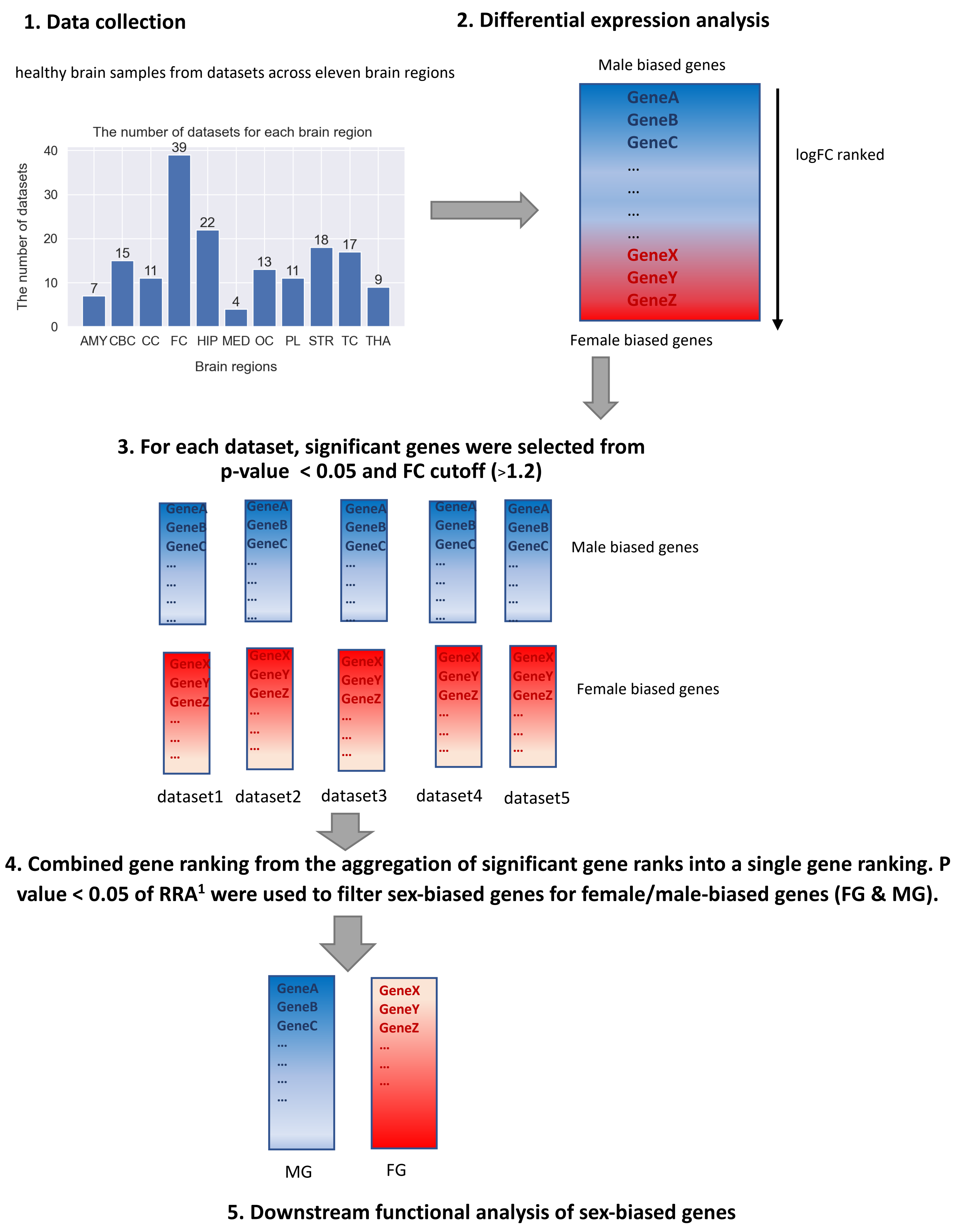

Figure S1. Schematic diagram of the workflow including rank aggregation for sex-biased genes detection in this study

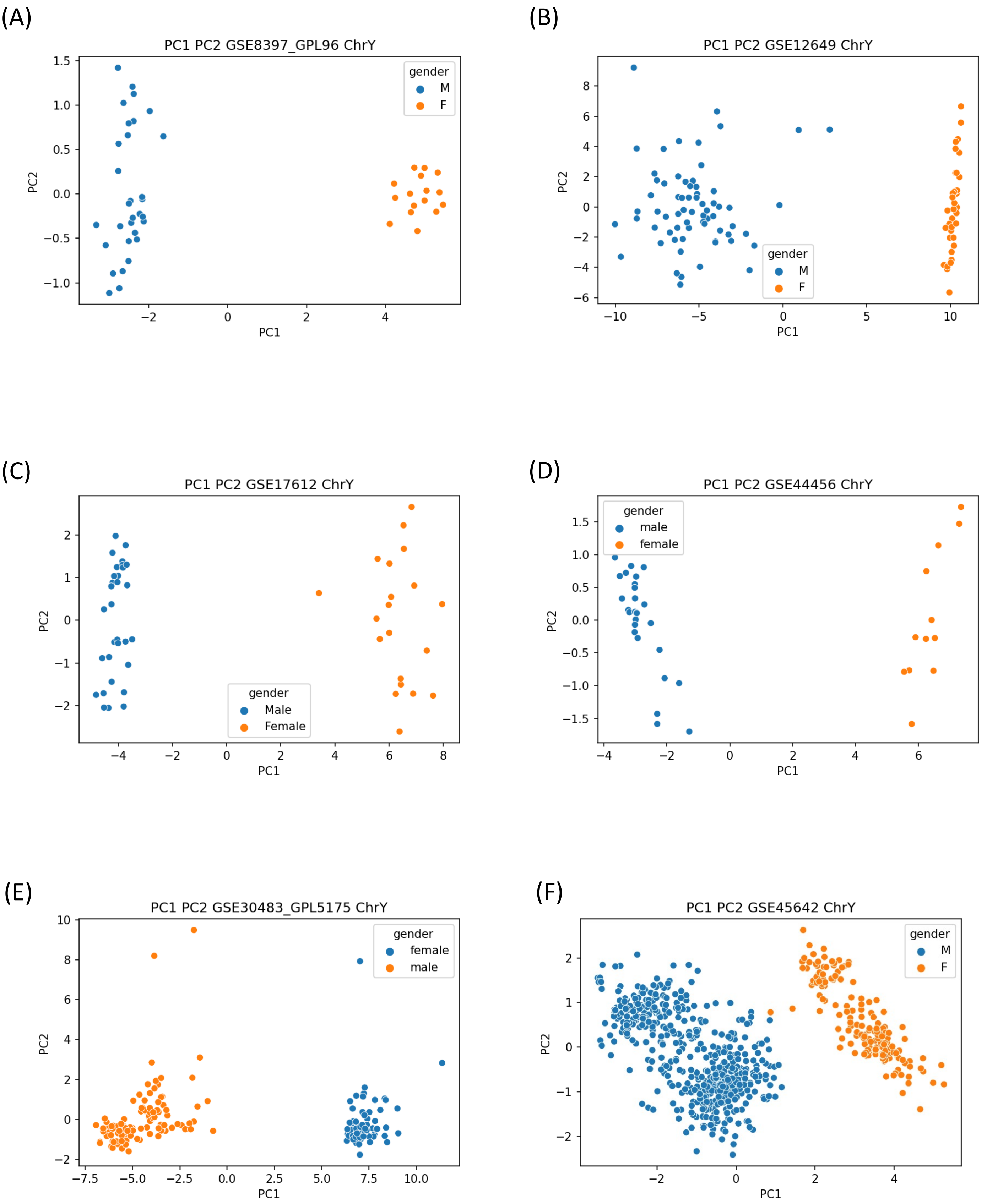

Figure S2. Principle component analysis (PCA) plot of Y- chromosome genes expression for GSE8397 (A) , GSE12649 (B), GSE17612 (C), GSE44456 (D), GSE30483 (E) and GSE45642 (F)

	GSE11882	GSE53890
Coefficient age-variable	$\|x\| > 0.75$	$\|x\| >0.5$
Coefficient sex-variable	$\|x\| > 1.2.$	$\|x\| > 0.5$

Figure S3. The cutoff for coefficient of age and sex variables in section multiple regression analysis with age and sex as independent variable

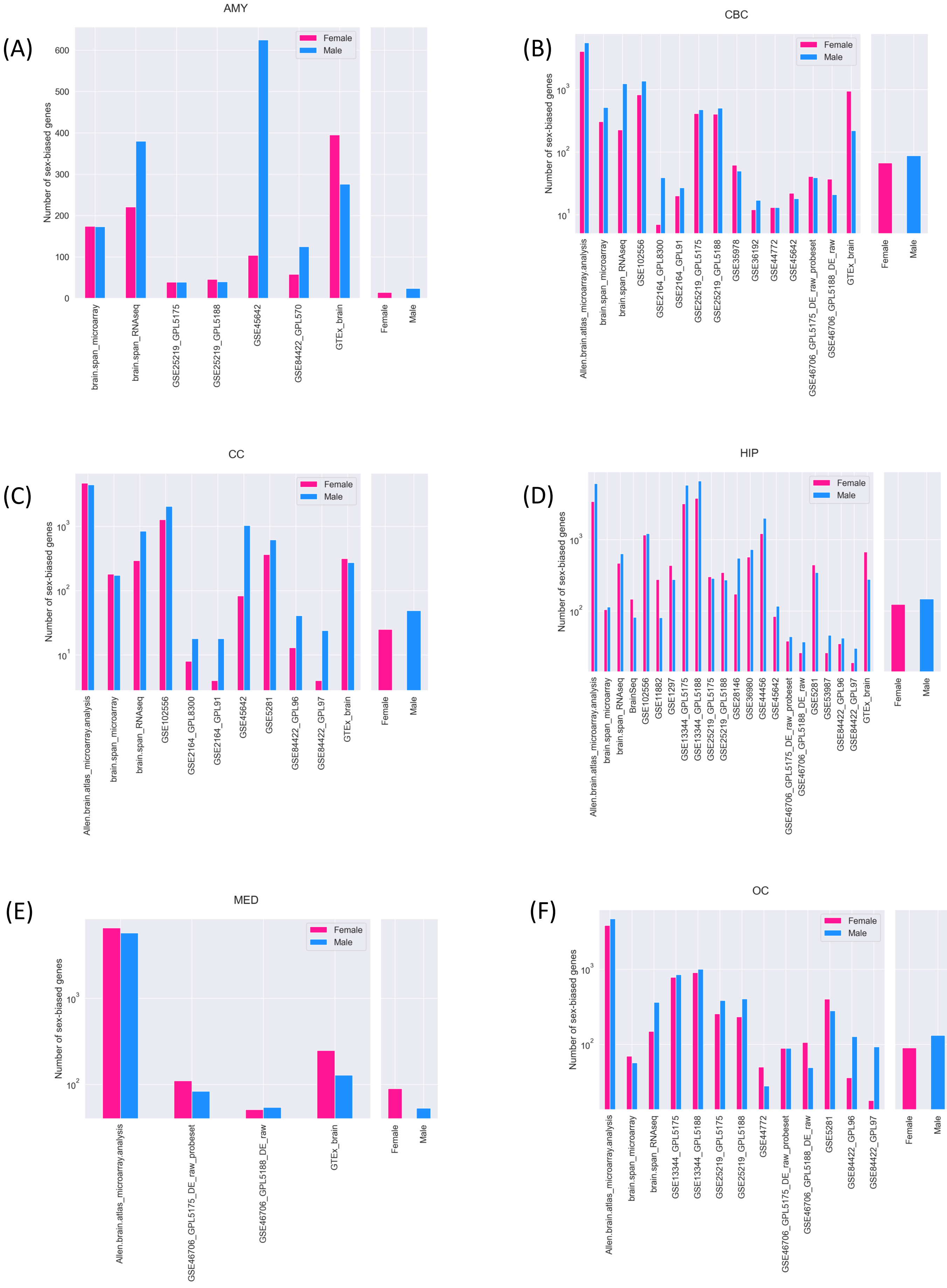

Figure S4. The number of sex-biased genes in individual datasets, aggregated lists in AMY, CBC, CC, HIP, MED and OC.

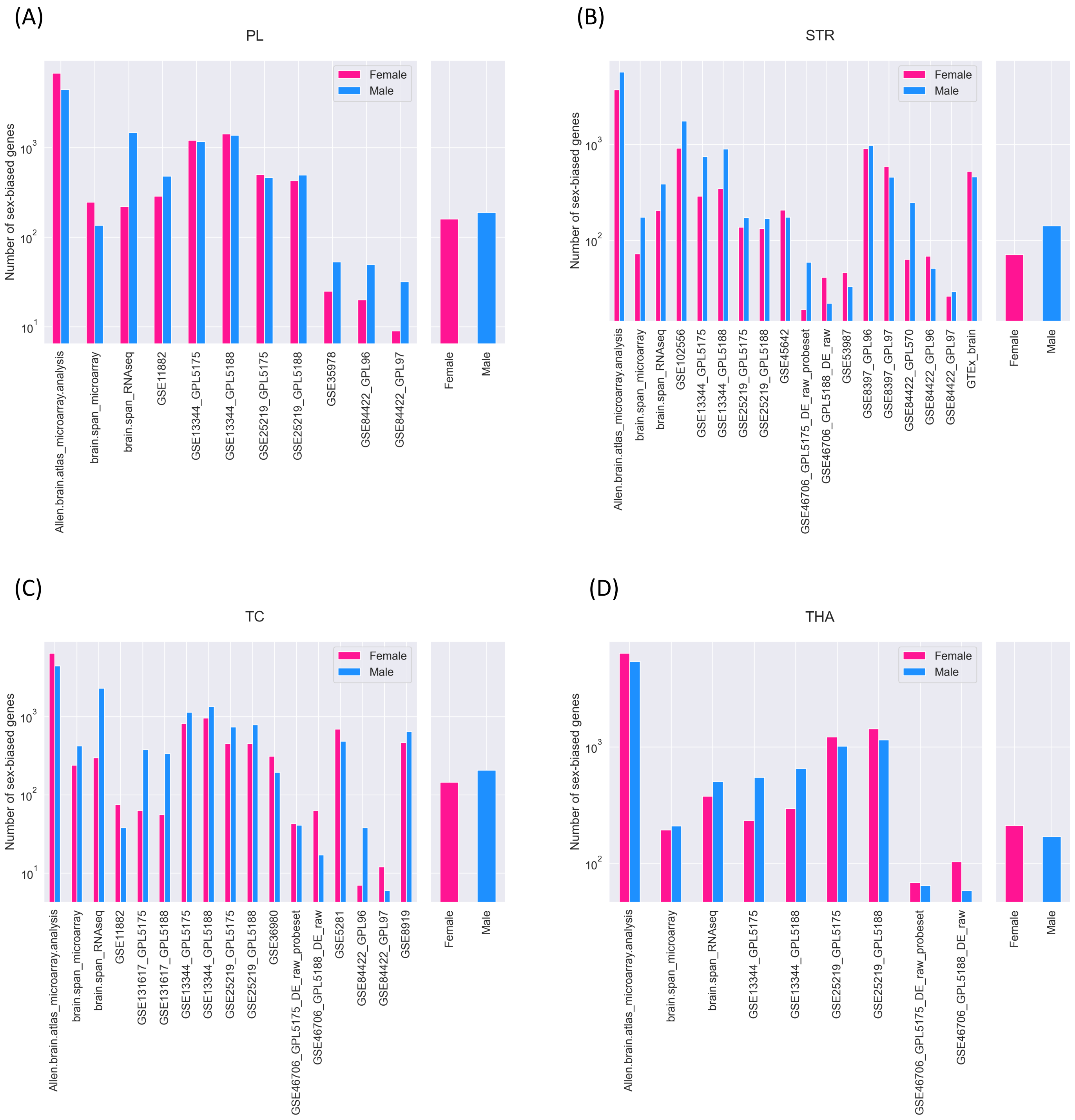

Figure S5. The number of sex-biased genes in individual datasets, aggregated lists in PL, STR, TC and THA.

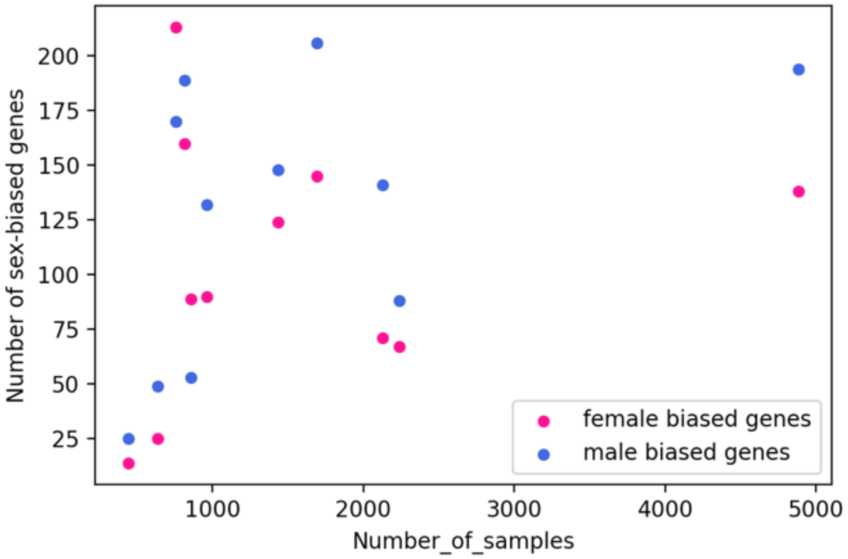

Figure S6. Plot of number of sex-biased genes in different number of brain samples.

(A)

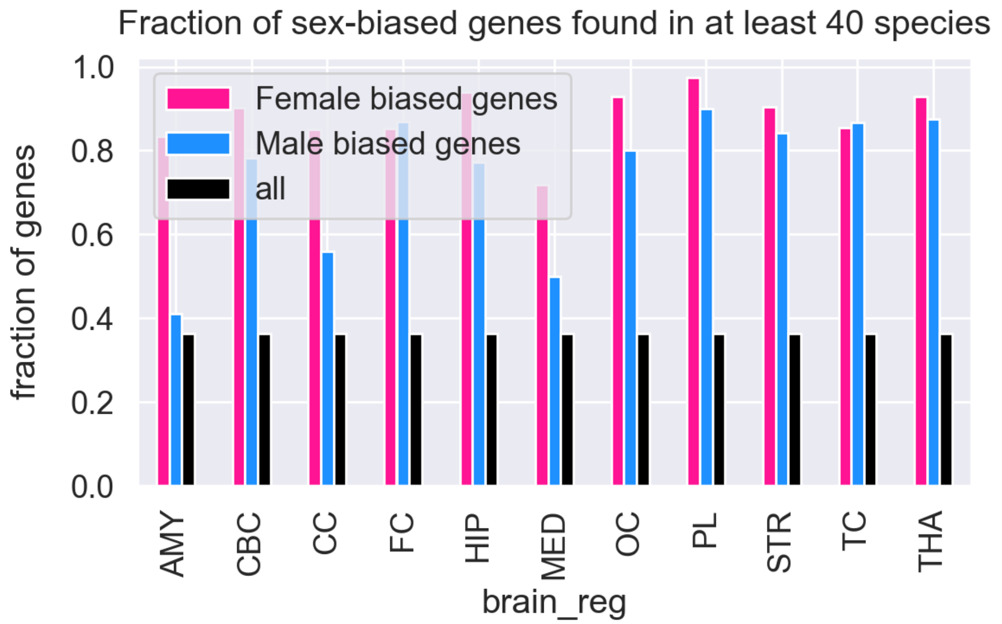

(B)

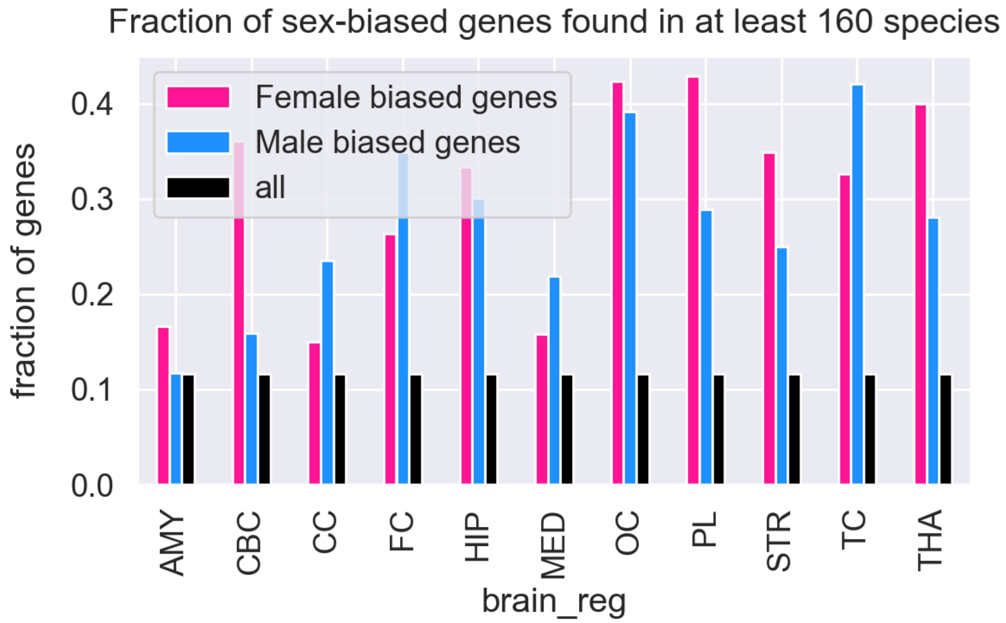

Figure S7. Fraction of sex-biased genes found at least in 40 species (A) and 160 species (B).

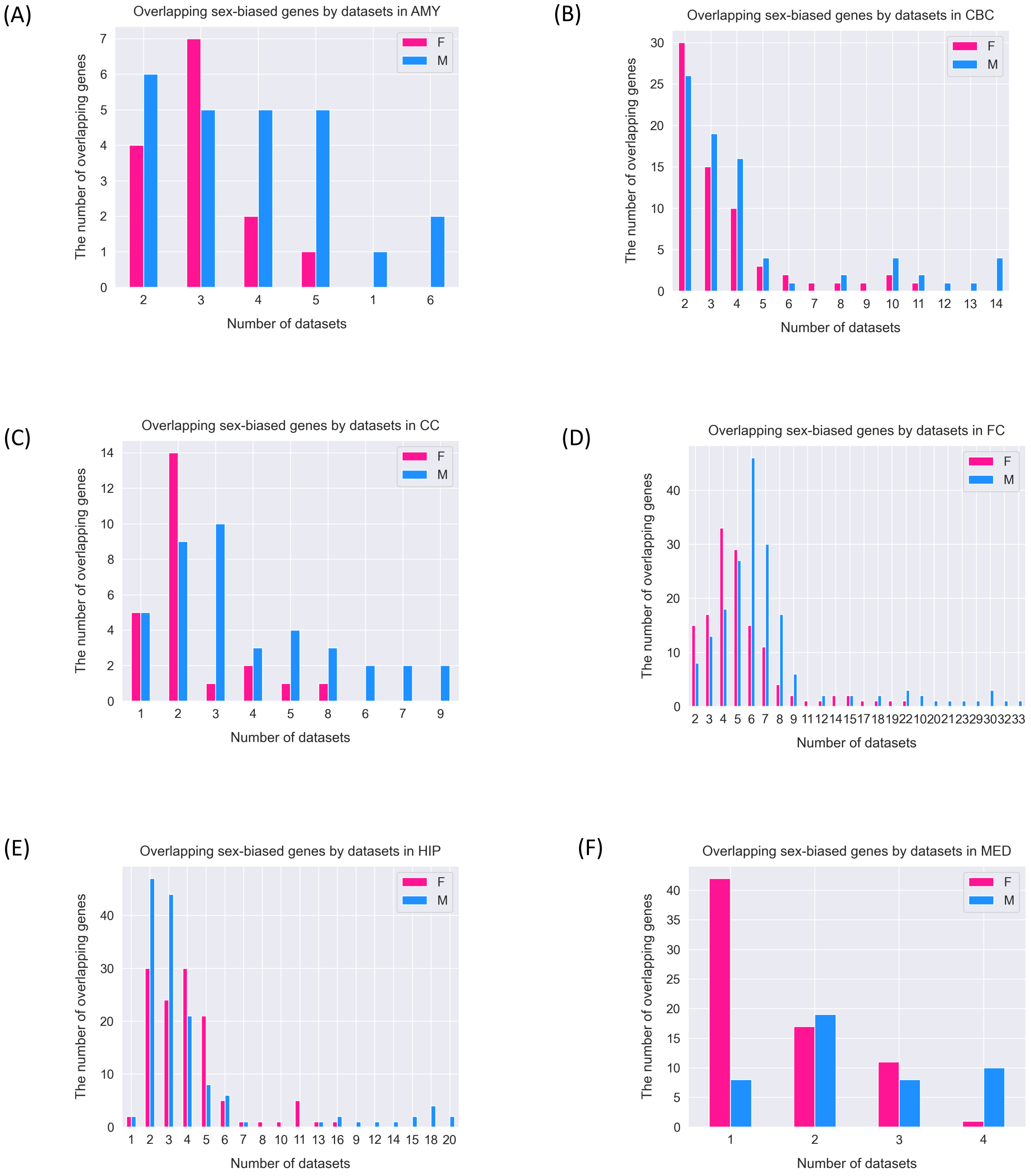

Figure S8. The number of overlapping sex-biased genes by number of datasets for AMY, CBC, CC, FC, HIP and MED

(A)

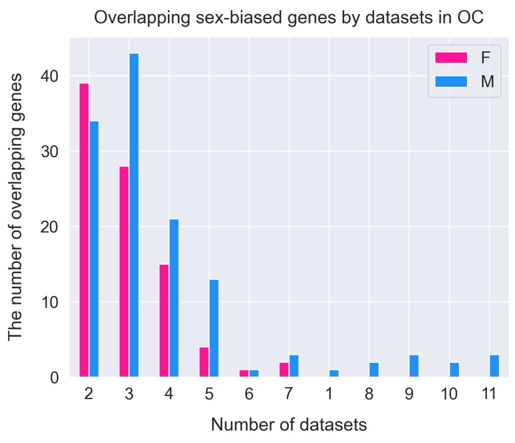

(B)

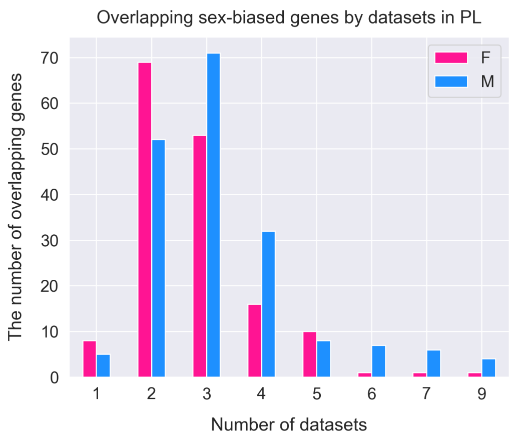

(C)

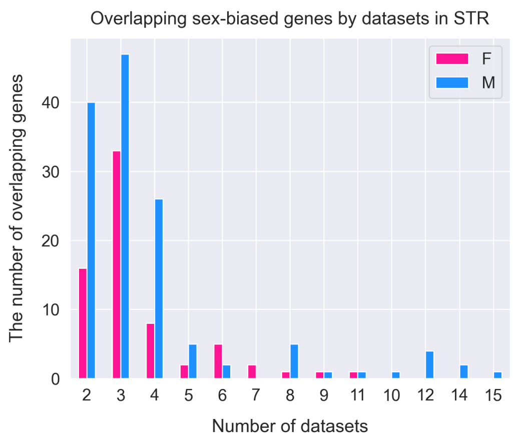

(D)

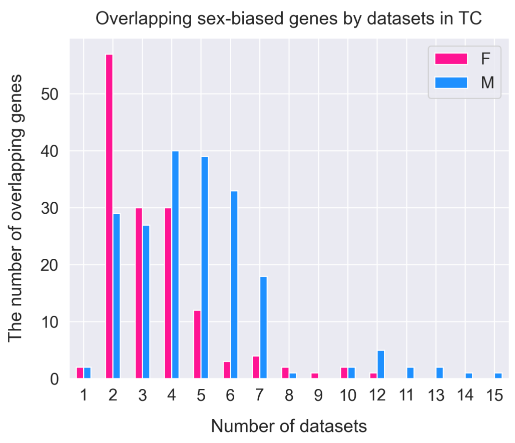

(E)

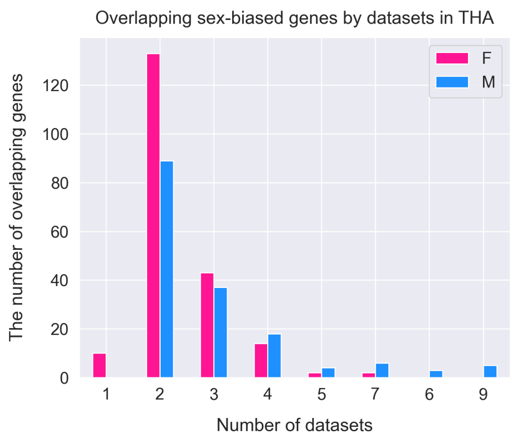

Figure S9. The number of overlapping sex-biased genes by number of datasets for OC, PL, STR, TC and THA

(A)

(B)

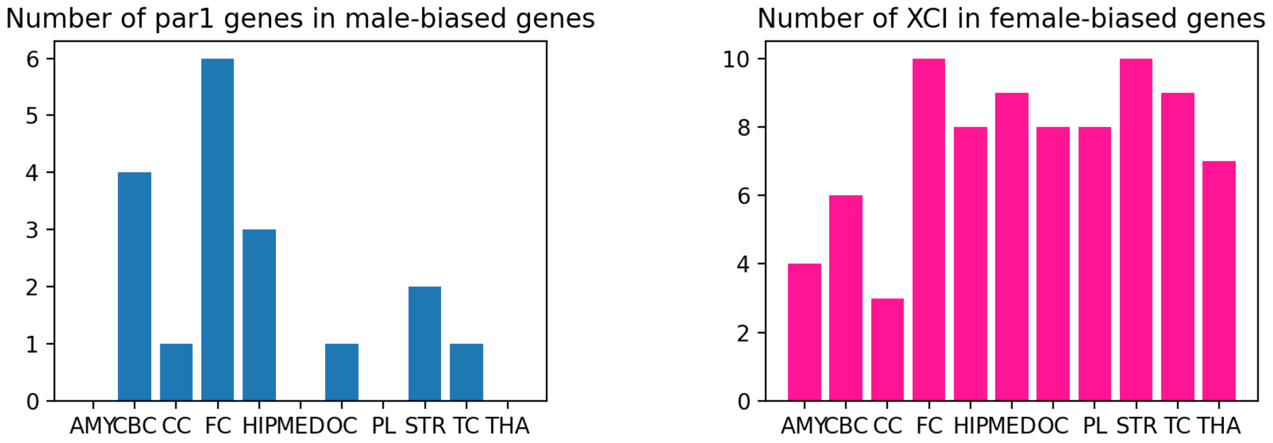

Figure S10. The number of par1 genes in male-biased genes (A) and the number of XCI female-biased genes (B). XCI gene list was from Chen et al. (2016)[2].

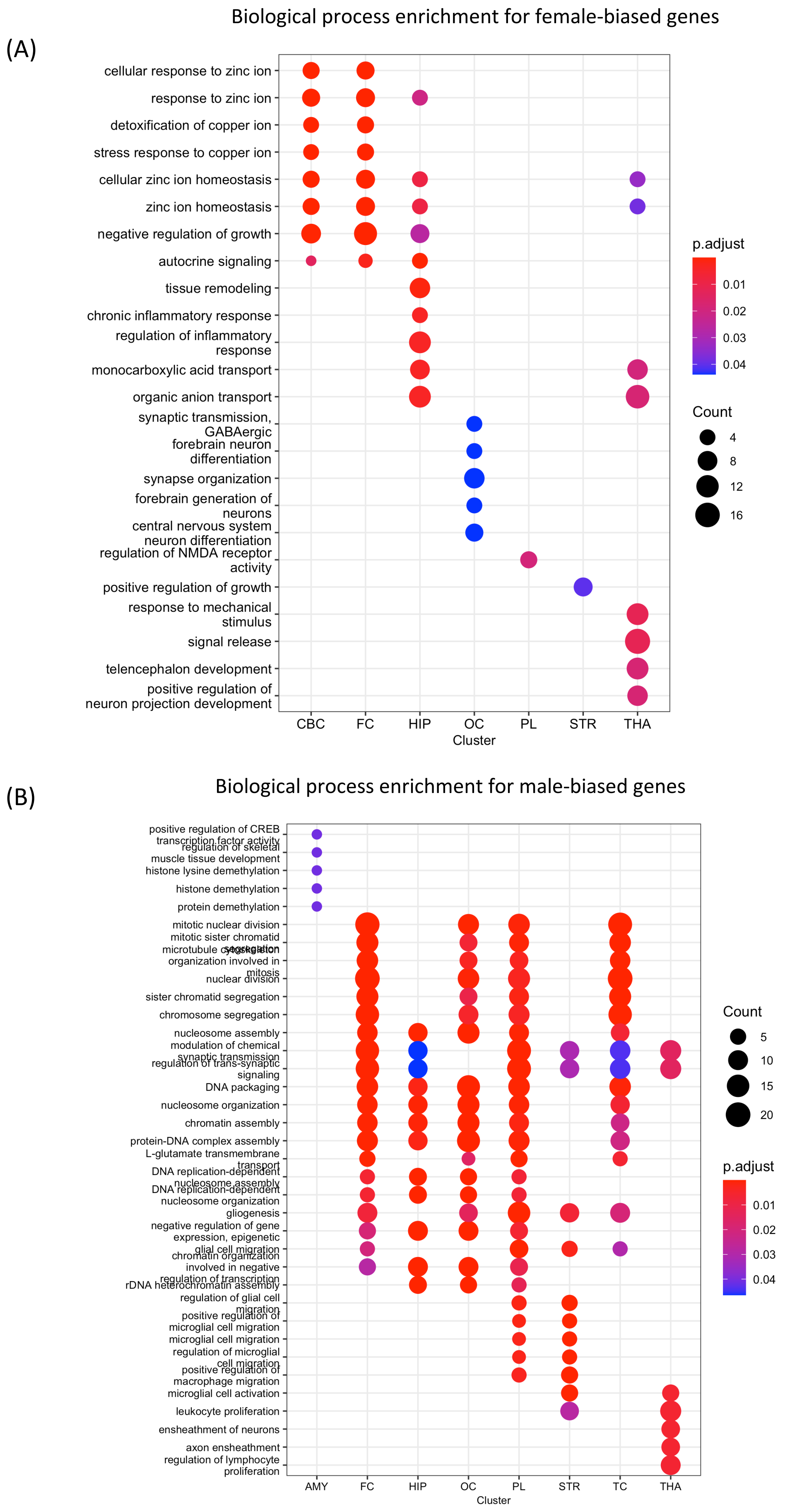

Figure S11. Gene ontology enrichment analysis of sex-biased genes for biological process. The top 5 enrich terms across brain regions in female-biased genes (A) and male-biased genes (B).

(A)

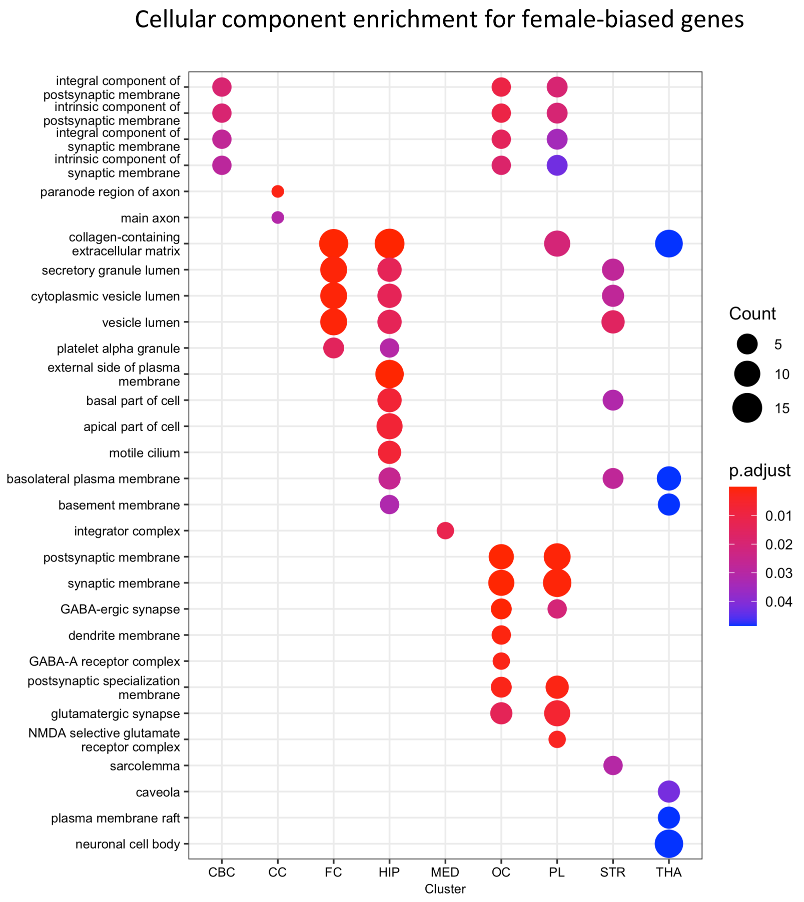

(B)

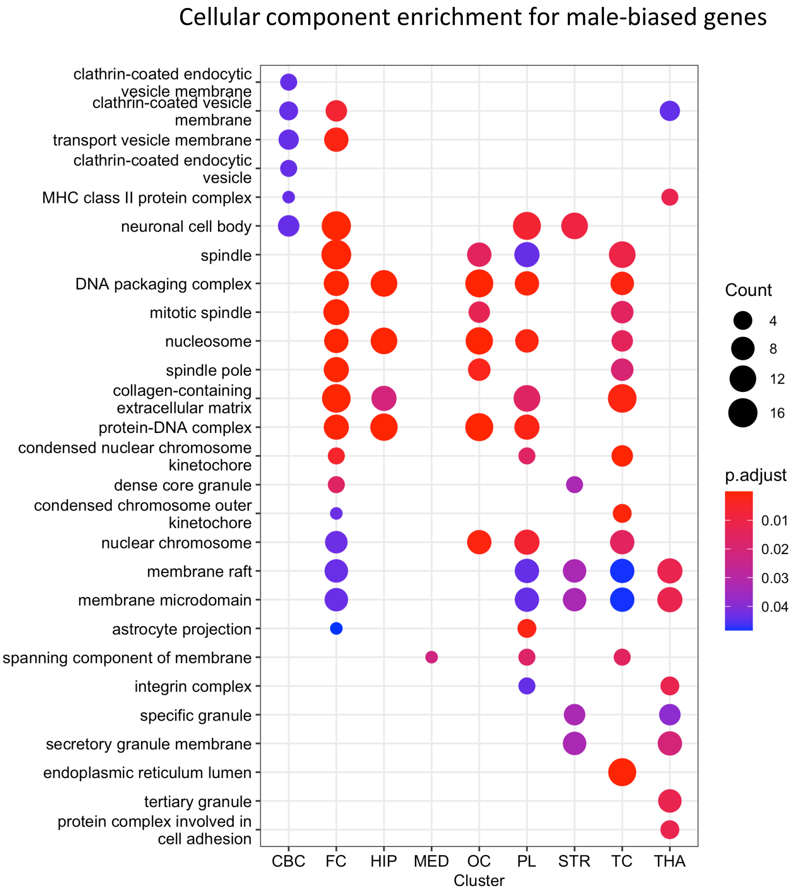

Fig S12. Gene ontology enrichment analysis of sex-biased genes for cellular component. The top 5 enrich terms across brain regions in female-biased genes (A) and male-biased genes (B).

(A)

Molecular function enrichment for female-biased genes

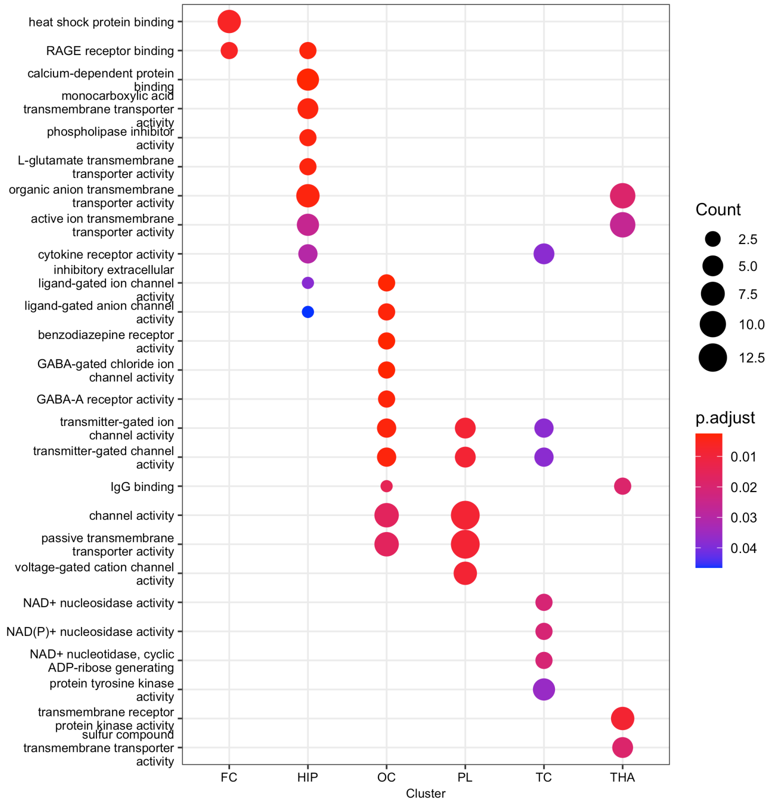

(B)

Molecular function enrichment for male-biased genes

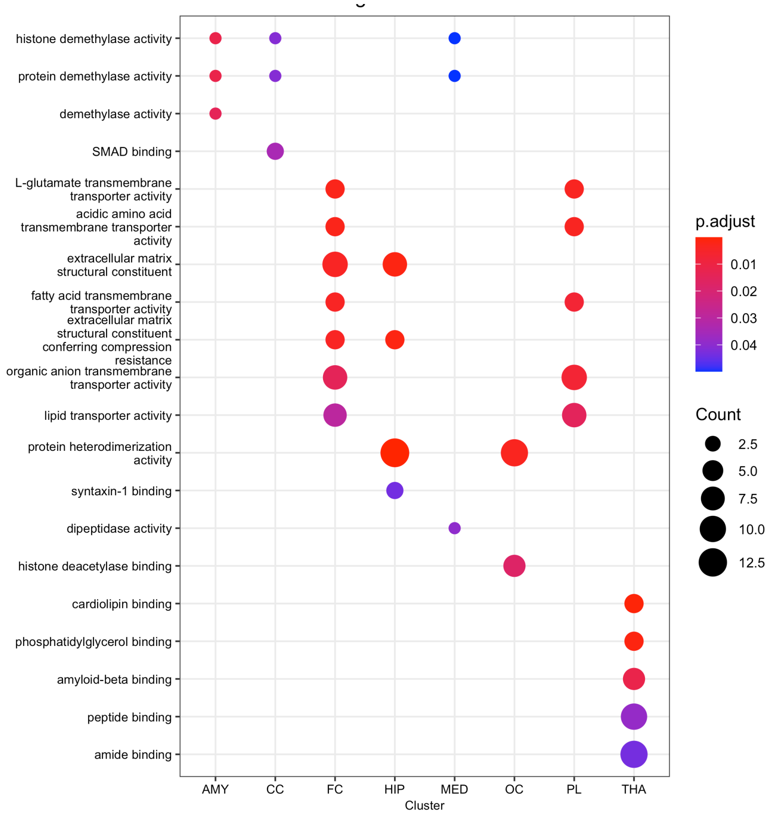

Fig S13. Gene ontology enrichment analysis of sex-biased genes for molecular functions. The top 5 enrich GO terms across brain regions in female-biased genes (A) and male-biased genes (B)

(A)

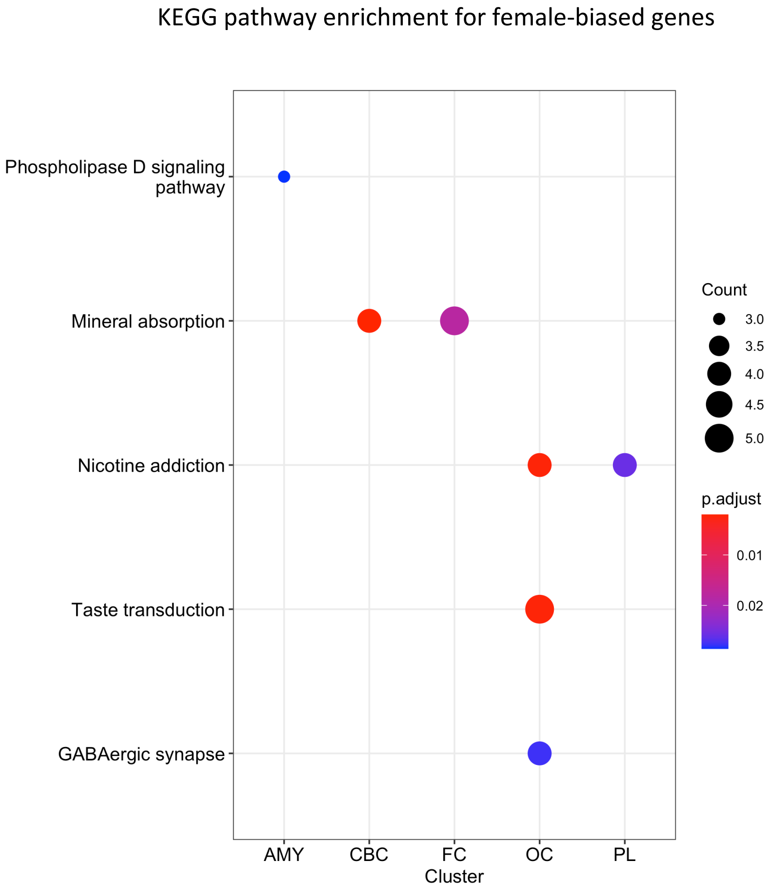

(B)

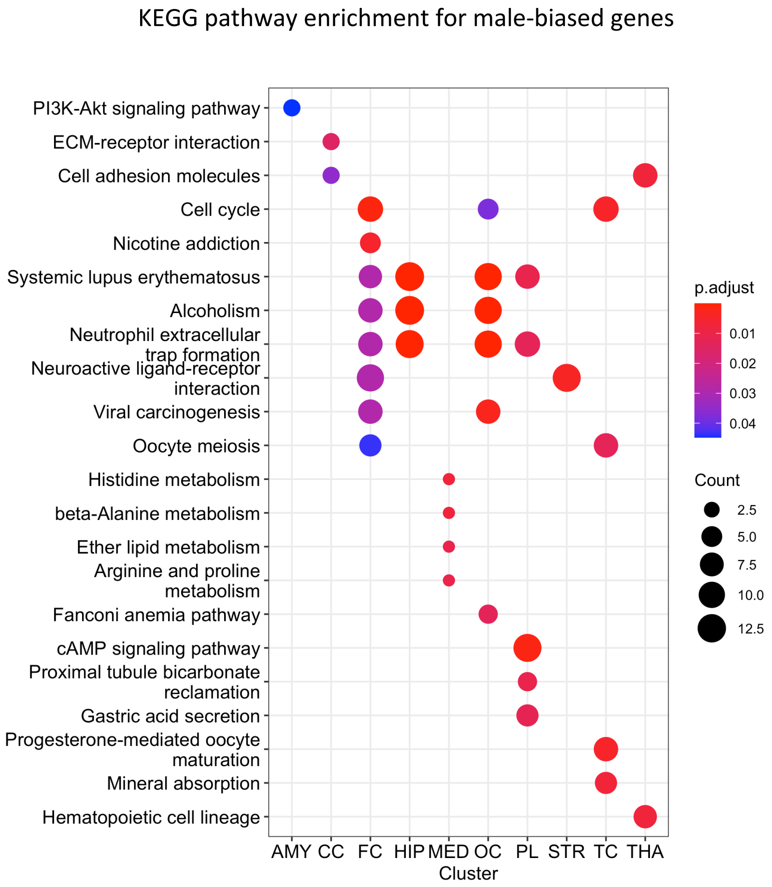

Fig S14. KEGG pathway enrichment analysis of sex-biased genes. The top 5 enrich GO terms across brain regions in female-biased genes (A) and male-biased genes (B)

(A)

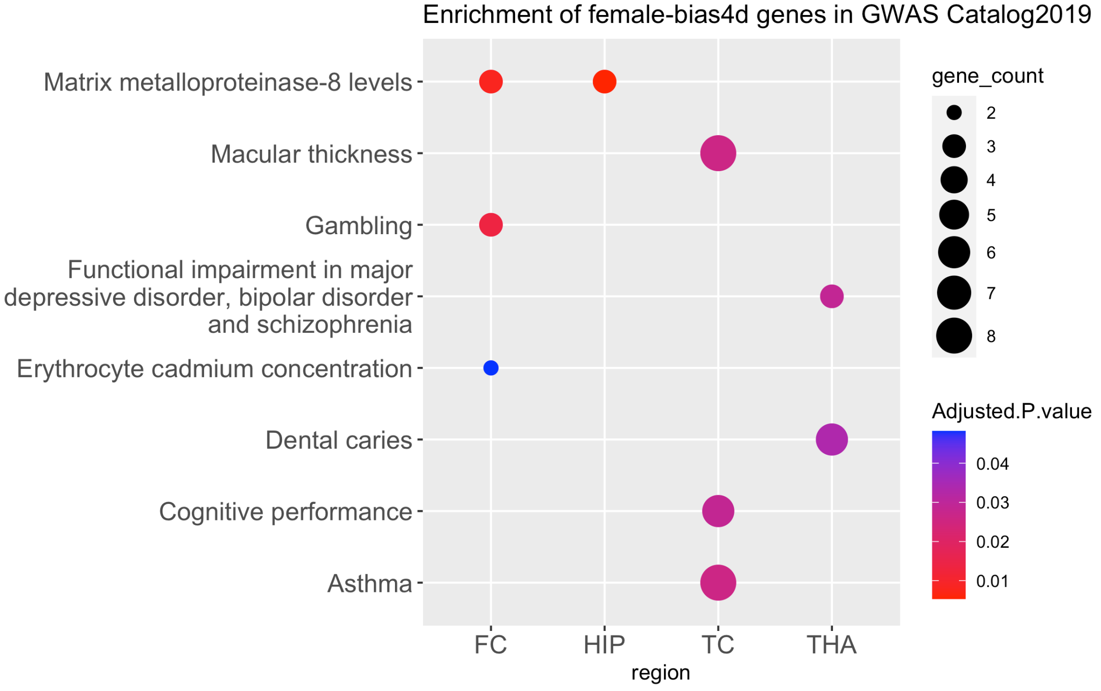

(B)

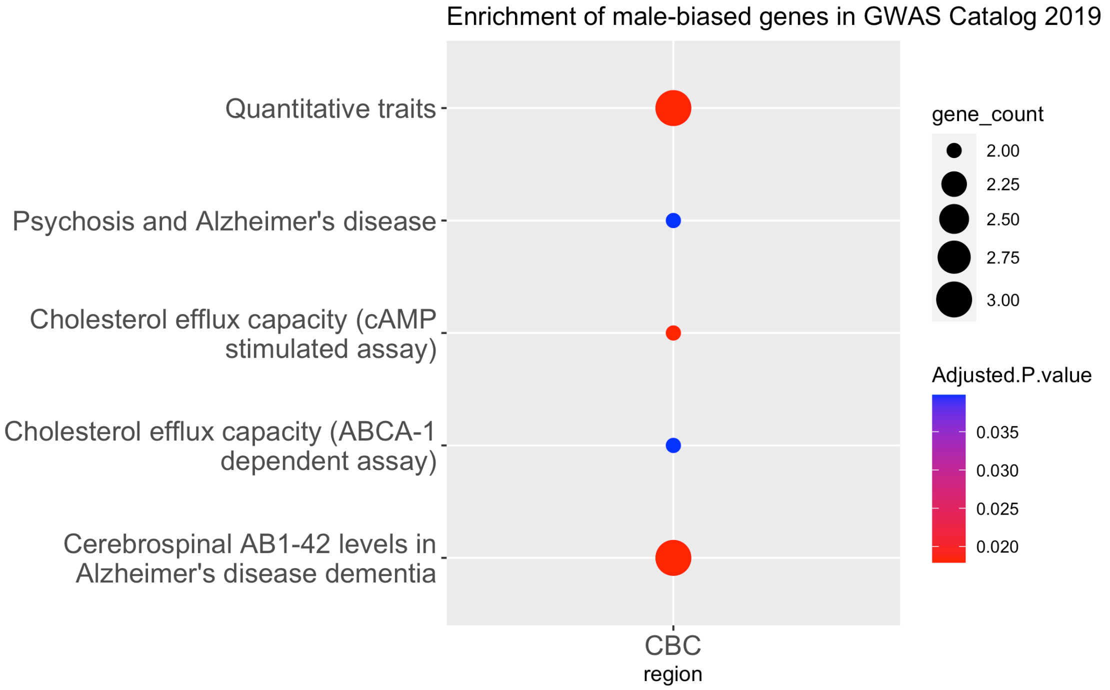

Fig S15. GWAS catalog 2019 enrichment across brain regions in female-biased genes (A) and male-biased genes (B)

(A)

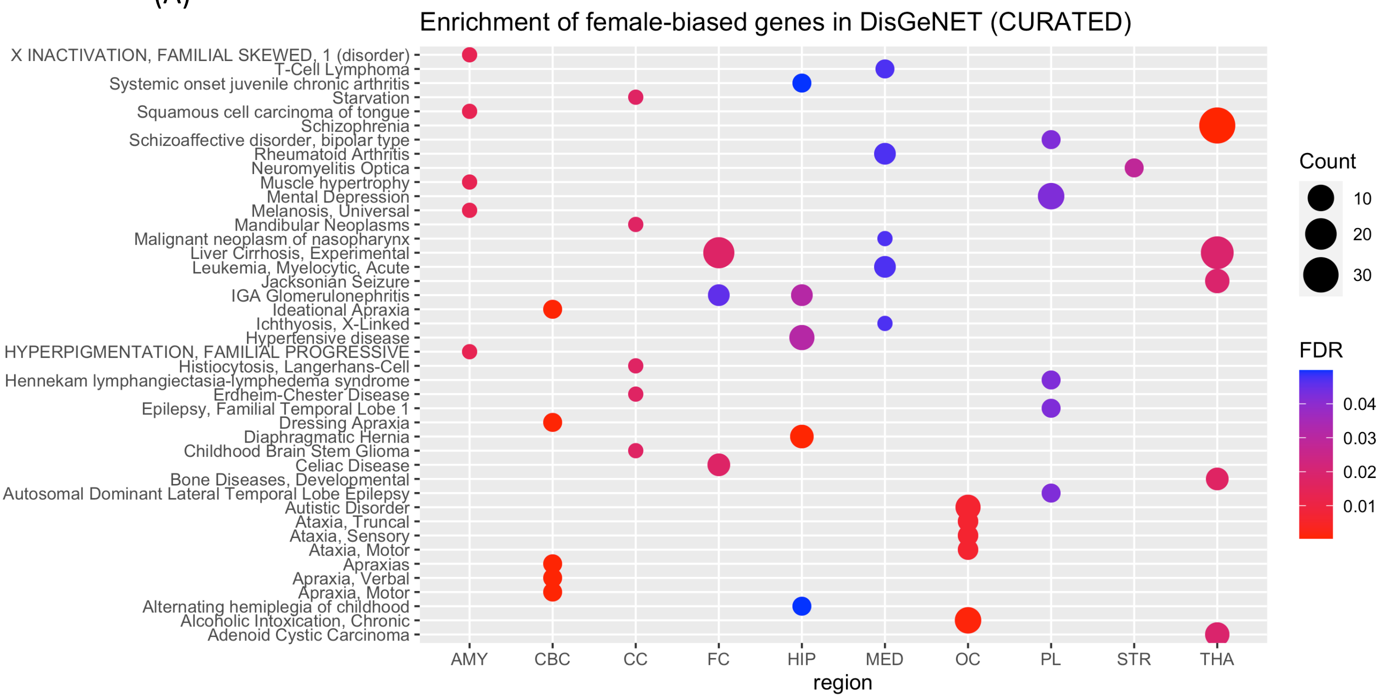

(B)

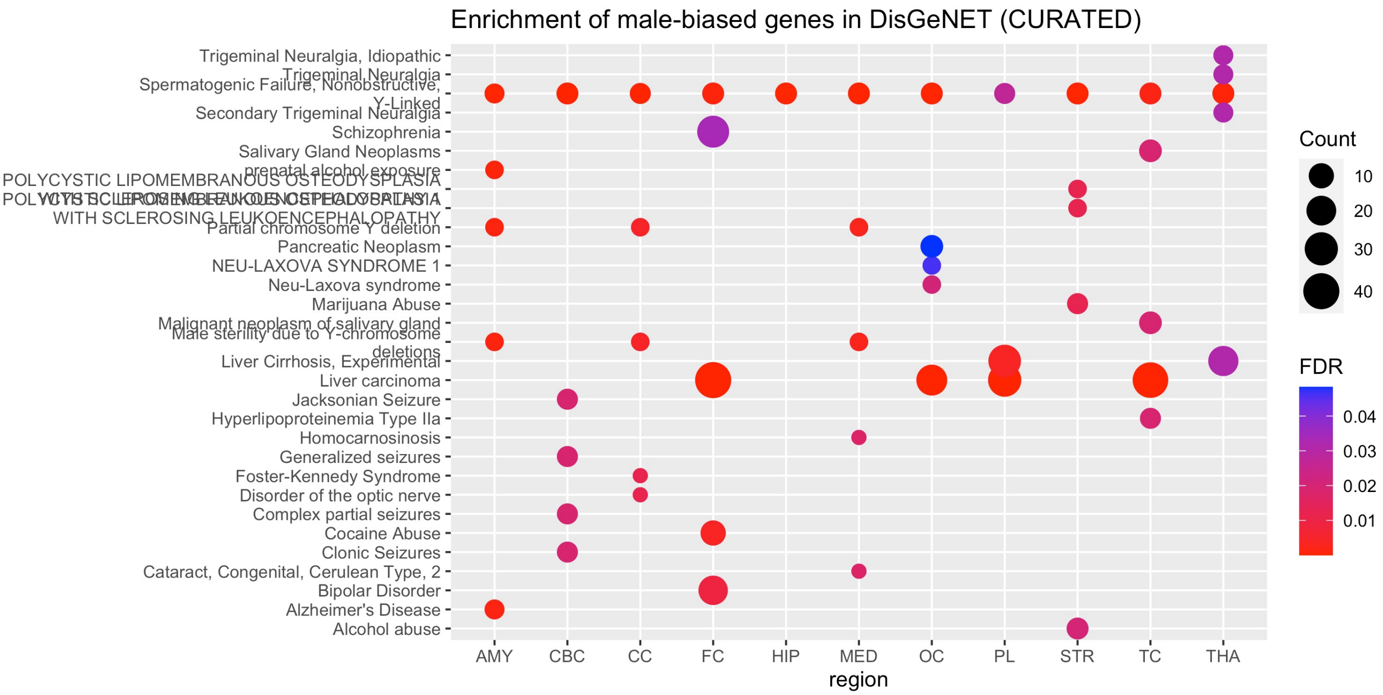

Fig S16. DisGeNET enrichment analysis of sex-biased genes across brain regions in female-biased genes (A) and male-biased genes (B)

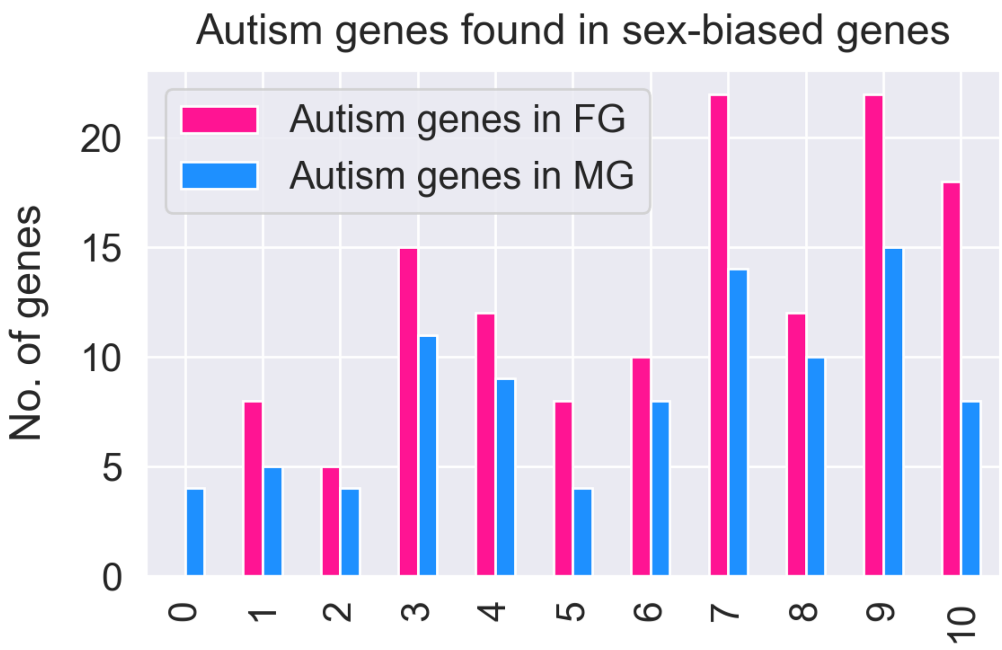

Fig S17. The number of autism genes found in sex-biased genes. Autism genes come from SFARI database (https://gene.sfari.org/).

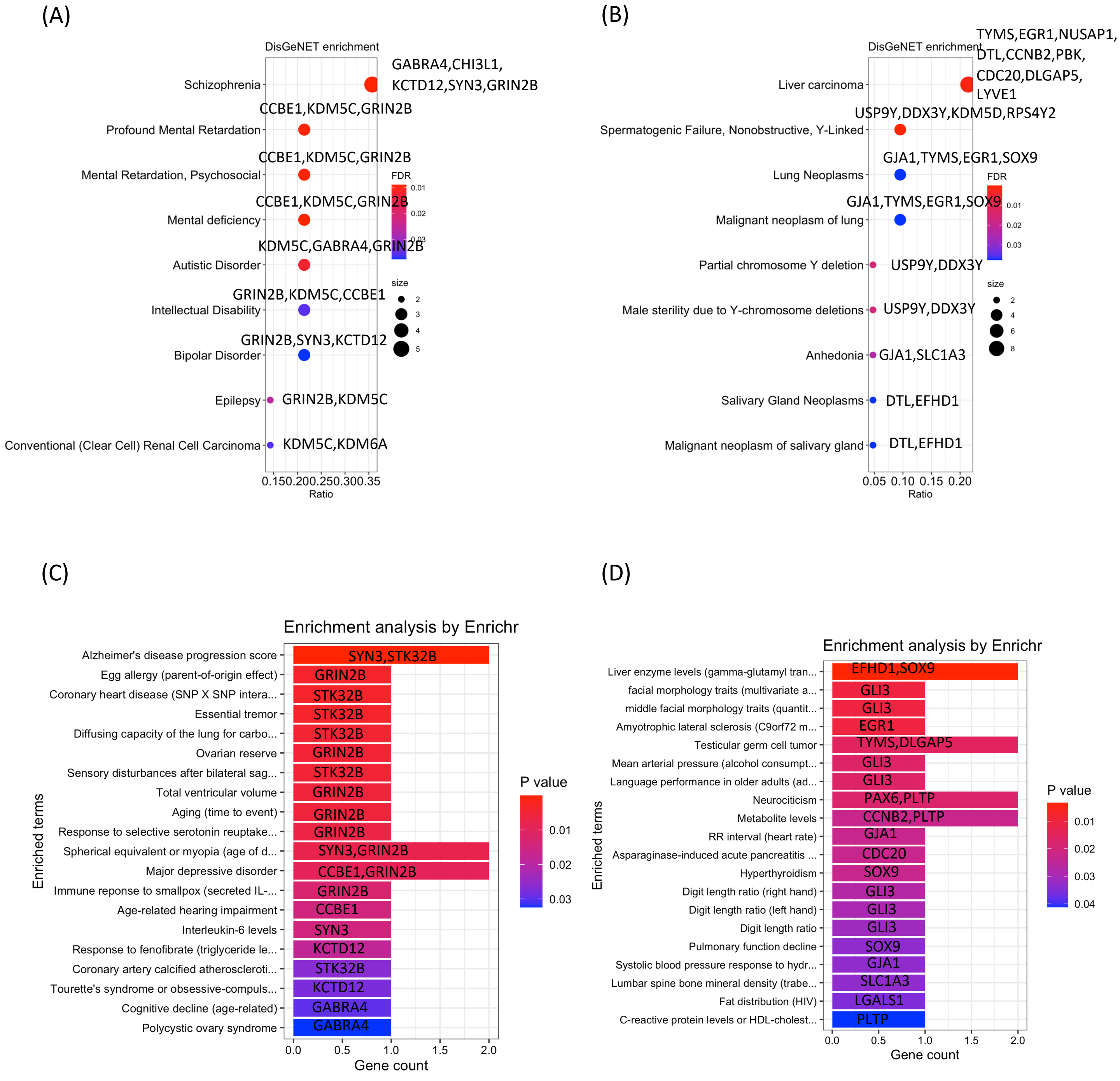

Fig S18. DisGeNet (CURATED) enrichment of overlap sex-biased genes across FC,PL,TC and OC in female-biased genes (A) and male-biasd genes (B). GWAS catalog 2019 enrichment analysis of overlap sex-biased genes across FC, PL, TC and OC in female-biased genes (C) and male-biased genes (D)

(A) Female overlap genes

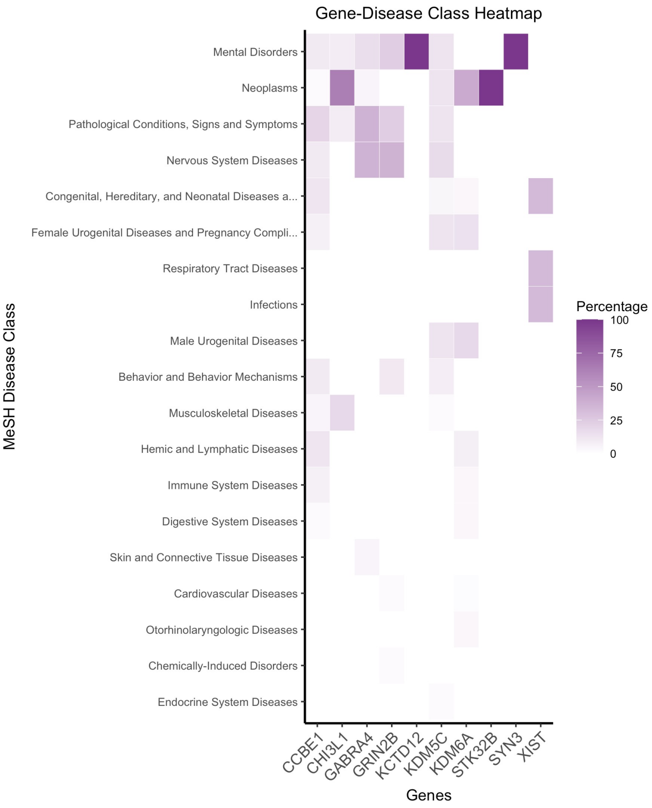

(B) male overlap genes

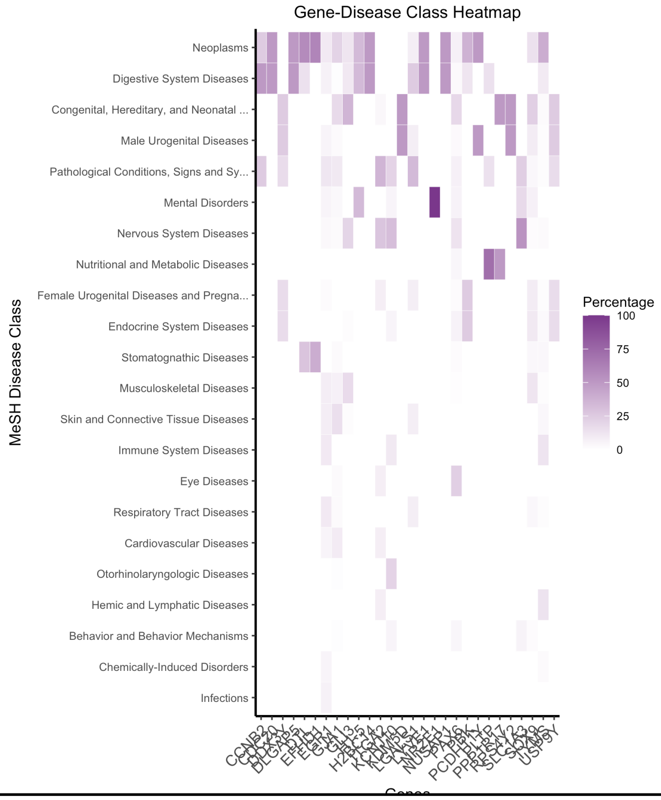

Fig S19. Gene-disease class heatmap of DisGeNET for overlap sex-biased gene lists of PL, FC, TC and OC. Diseases are grouped by the their MeSH disease classes. The color scale is related to the percentage of disease in each class.

(A) Female biased genes

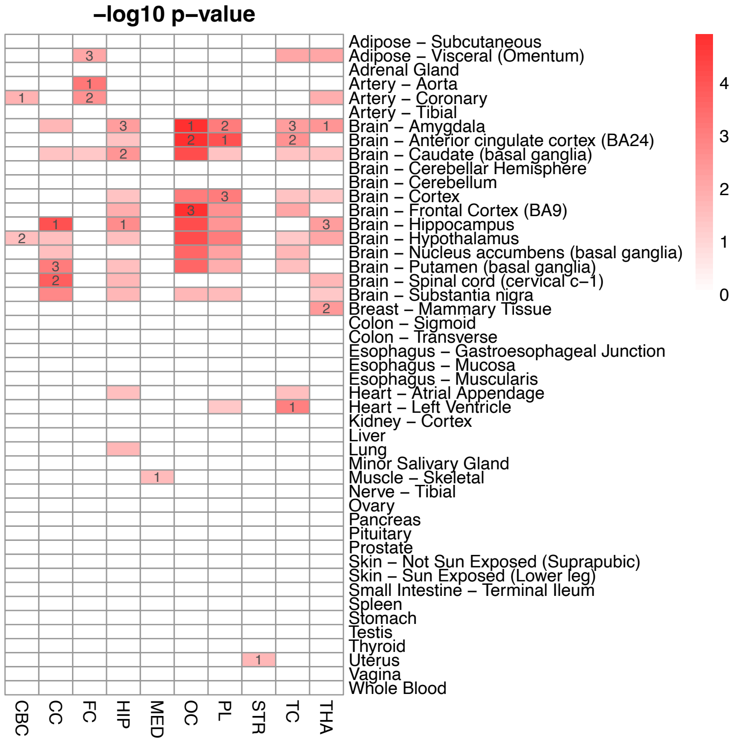

(B) Male biased genes

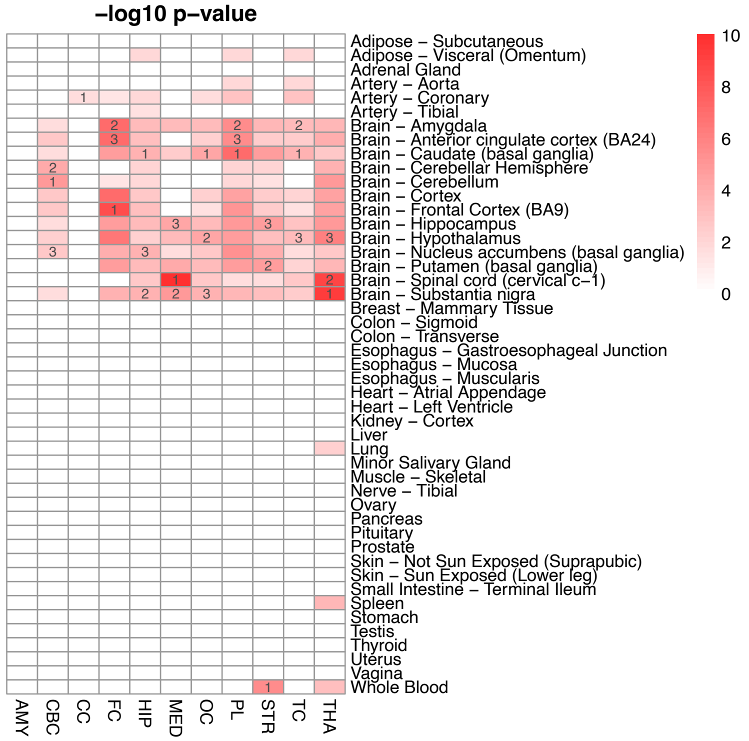

Fig S20. Tissue-specific enrichment analysis (TSEA) of female-biased genes (A) and male-biased genes (B)

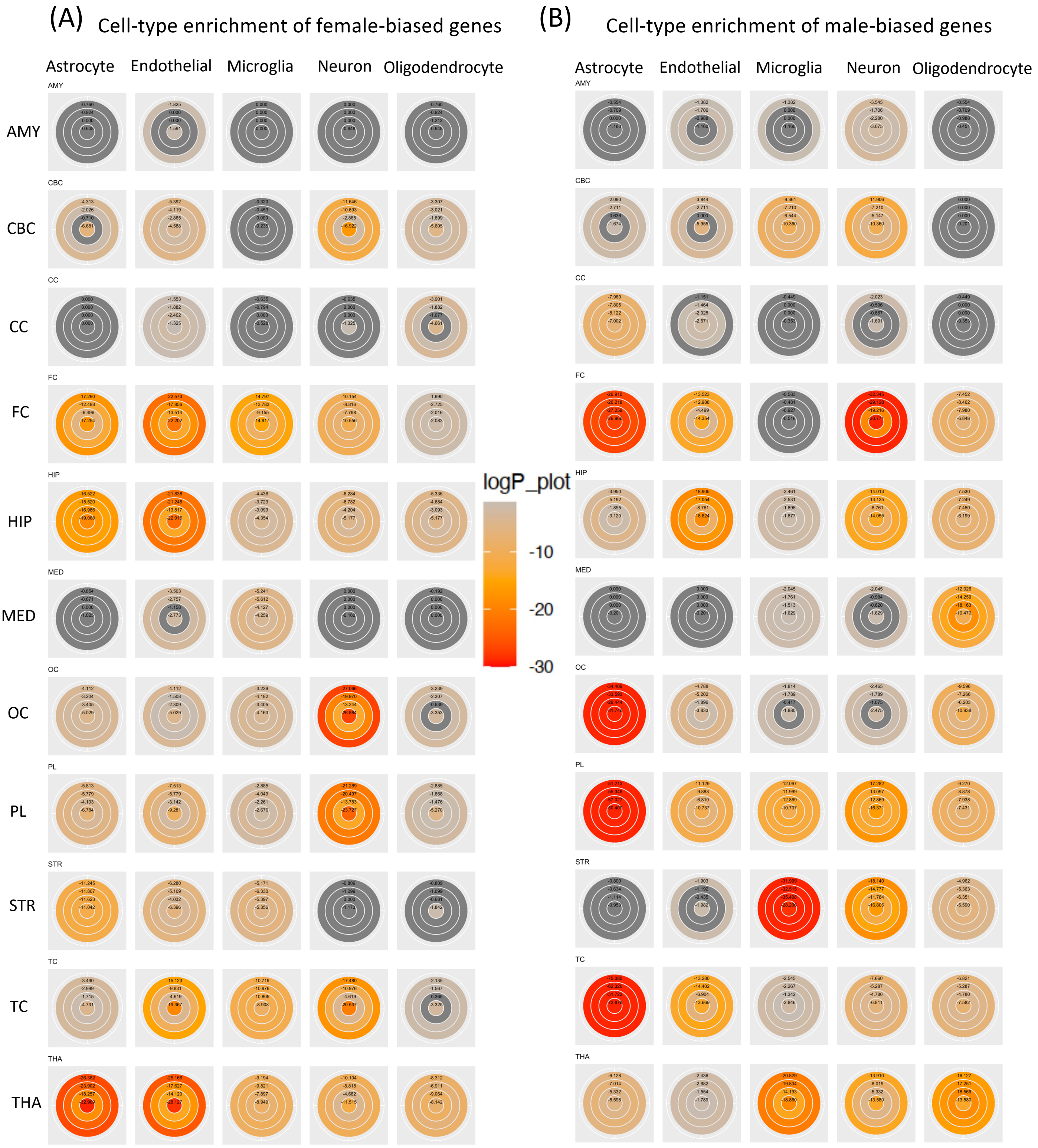

Fig S21. Cell type enrichment of sex-biased genes from McKenzie et al.[3] gene sets

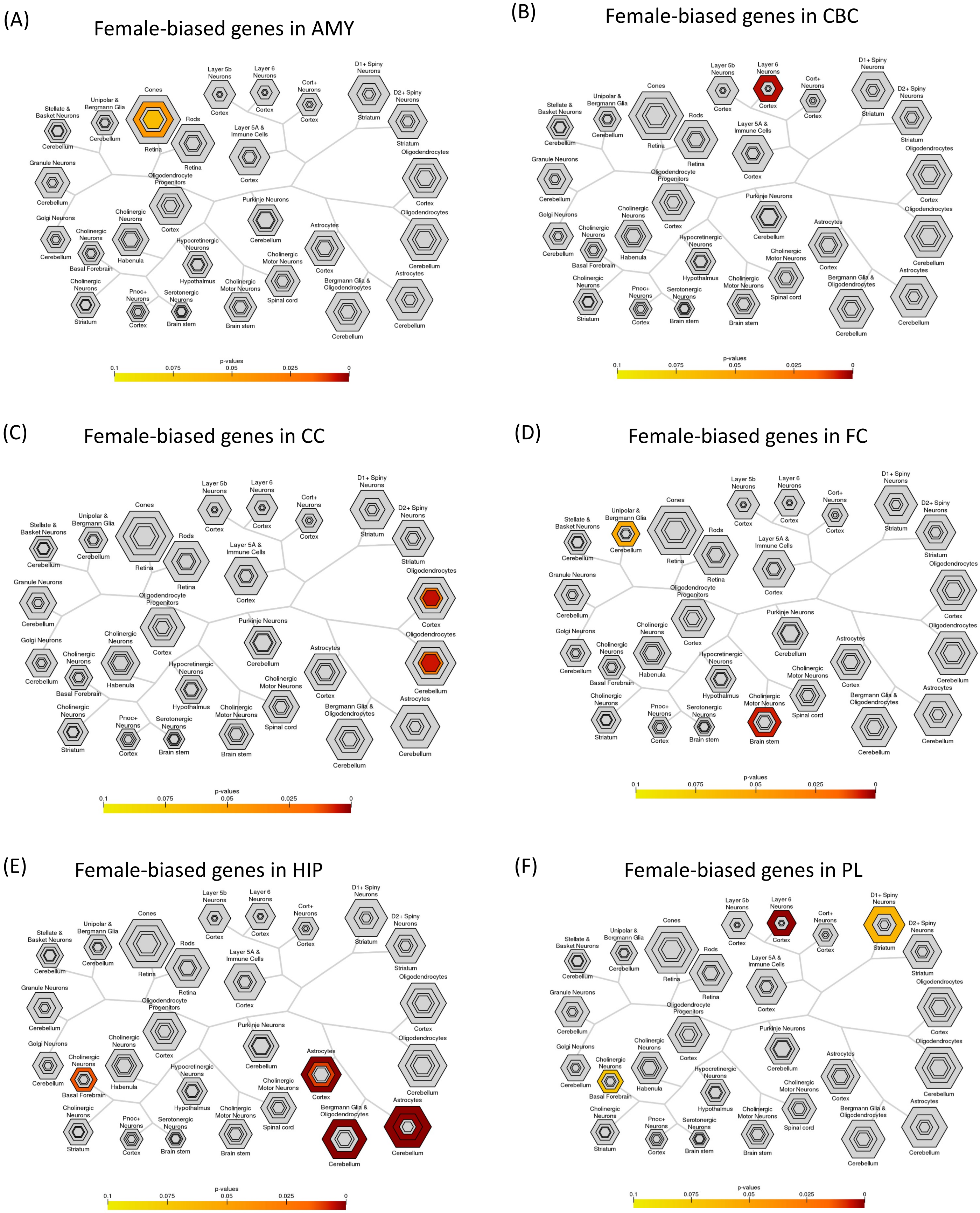

Fig S22. Cell type specific expression analysis (CSEA) of female baised genes in AMY, CBC, CC, FC, HIP and PL. The size of hexagon is represented the size of gene list with different of Specificity Index thresholds (pSI) varying from 0.05, 0.01, 0.001, 0.0001 and the color of hexagon is p-value for Fisher's Exact Test for overlap of cell type and sex-biased gene list. The plot is produced by CSEA tools from http://genetics.wustl.edu/jdlab/csea-tool-2/.

(A) Female-biased genes in STR

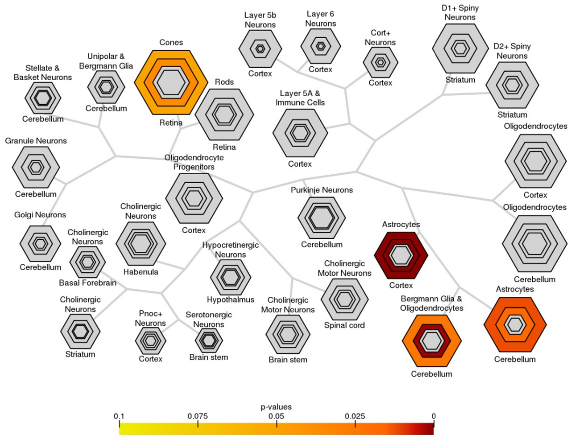

(B) Female-biased genes in TC

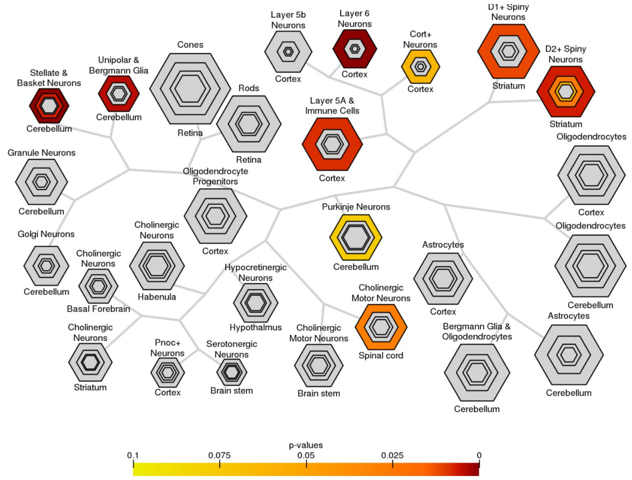

(C) Female-biased genes in THA

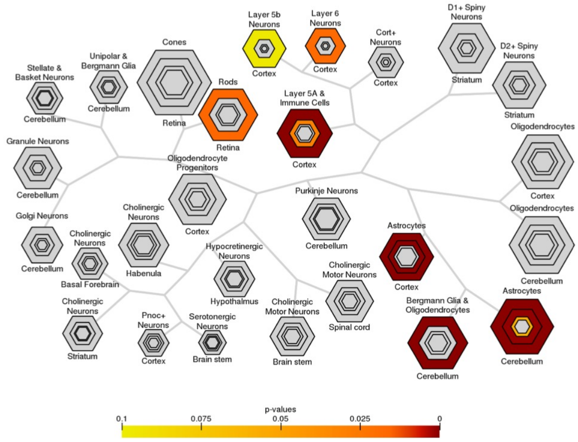

Fig S23. Cell type specific expression analysis (CSEA) of female baised genes in STR, TC and THA. The size of hexagon is represented the size of gene list with different of Specificity Index thresholds (pSI) varying from 0.05, 0.01, 0.001, 0.0001 and the color of hexagon is p-value for Fisher's Exact Test for overlap of cell type and sex-biased gene list. The plot is produced by CSEA tools from http://genetics.wustl.edu/jdlab/csea-tool-2/.

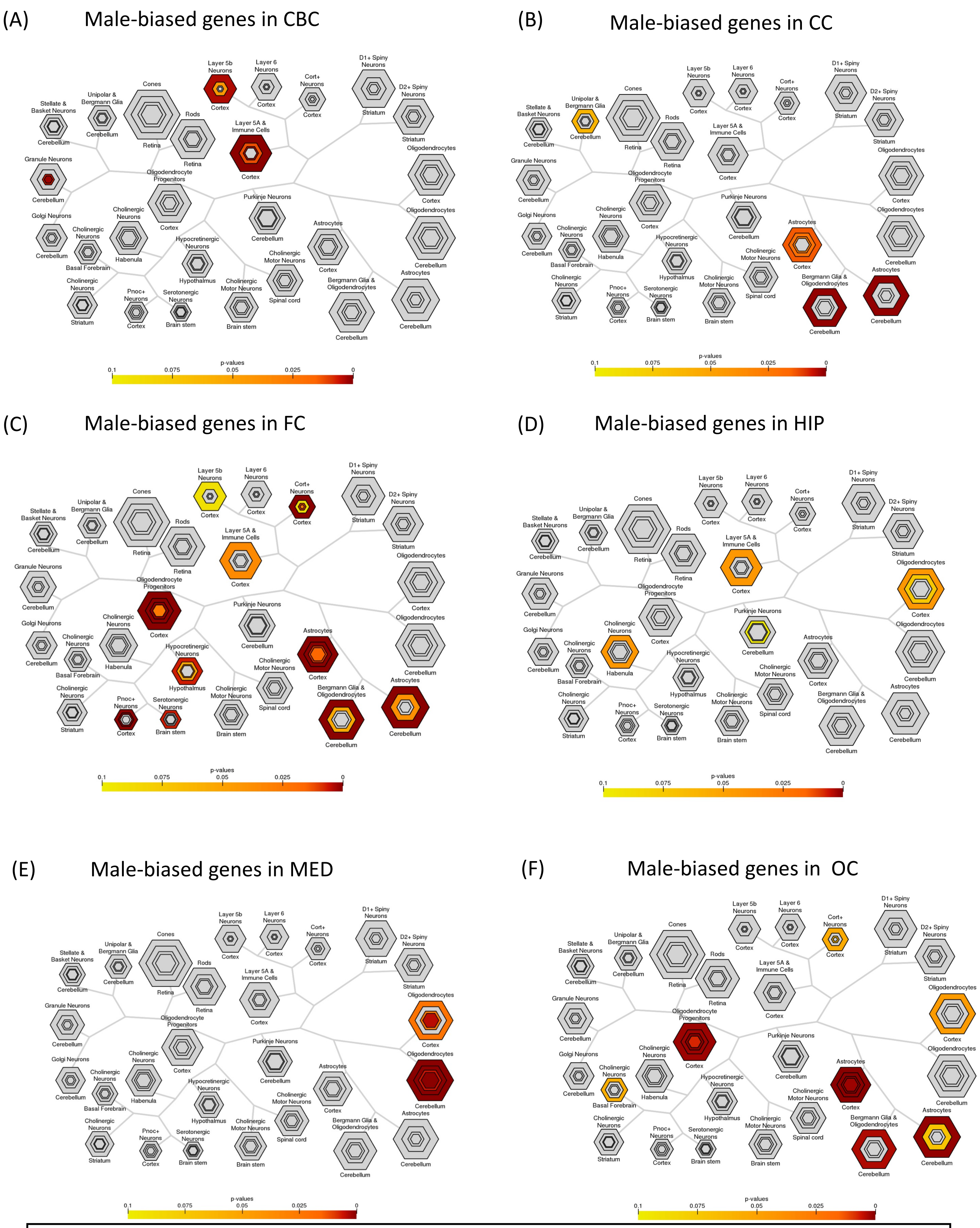

Fig S24. Cell type specific expression analysis (CSEA) of male baised genes in CBC, CC, FC, HIP, MED and OC. The size of hexagon is represented the size of gene list with different of Specificity Index thresholds (pSI) varying from 0.05, 0.01, 0.001, 0.0001 and the color of hexagon is p-value for Fisher's Exact Test for overlap of cell type and sex-biased gene list. The plot is produced by CSEA tools from http://genetics.wustl.edu/jdlab/csea-tool-2/.

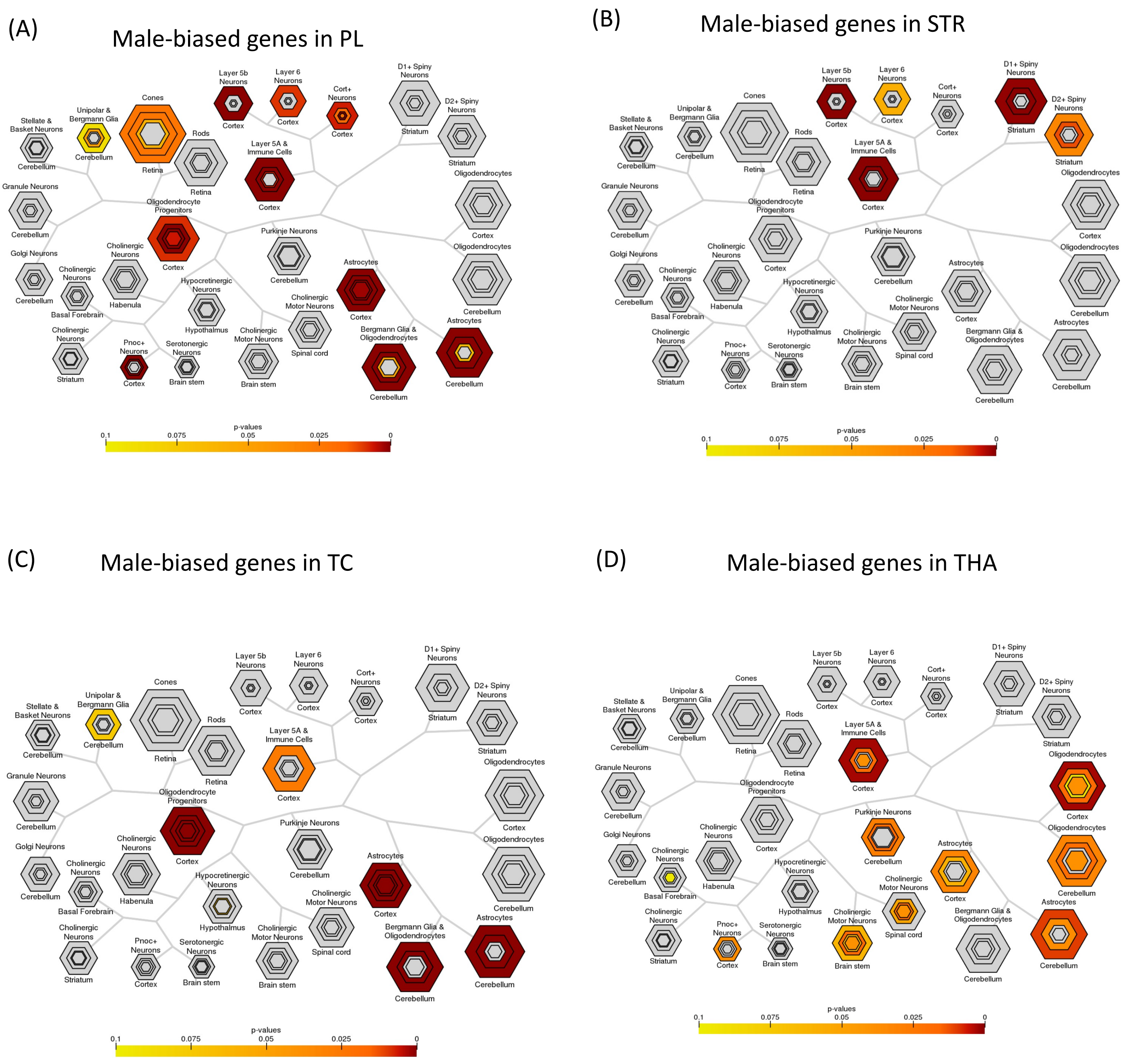

Fig S25. Cell type specific expression analysis (CSEA) of male baised genes in PL, STR, TC and THA. The size of hexagon is represented the size of gene list with different of Specificity Index thresholds (pSI) varying from 0.05, 0.01, 0.001, 0.0001 and the color of hexagon is p-value for Fisher's Exact Test for overlap of cell type and sex-biased gene list. The plot is produced by CSEA tools from http://genetics.wustl.edu/jdlab/csea-tool-2/.

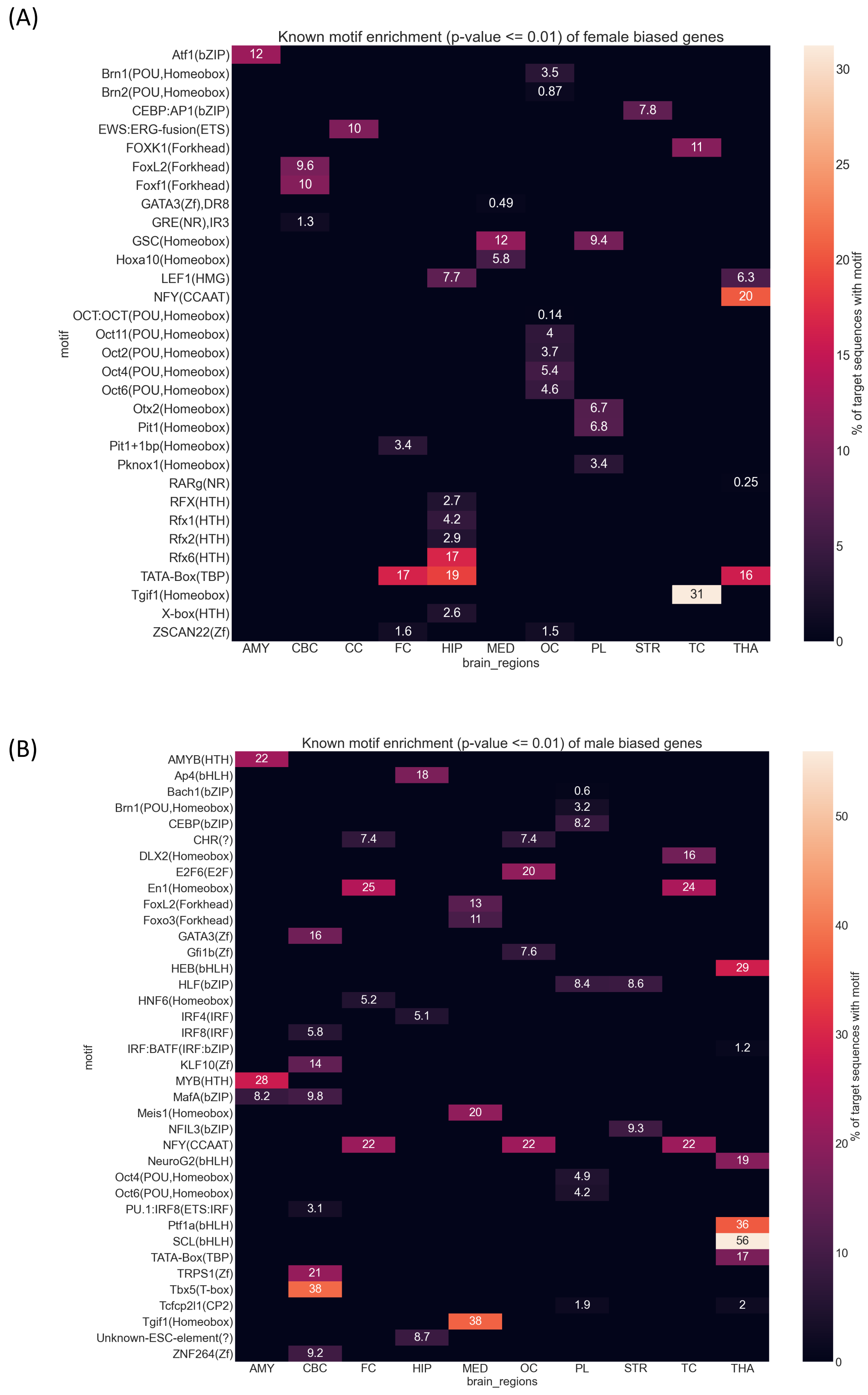

Fig S26. Known motif enrichment analysi by HOMER in the promoters of the female-biased genes (A) and male-biased genes (B)

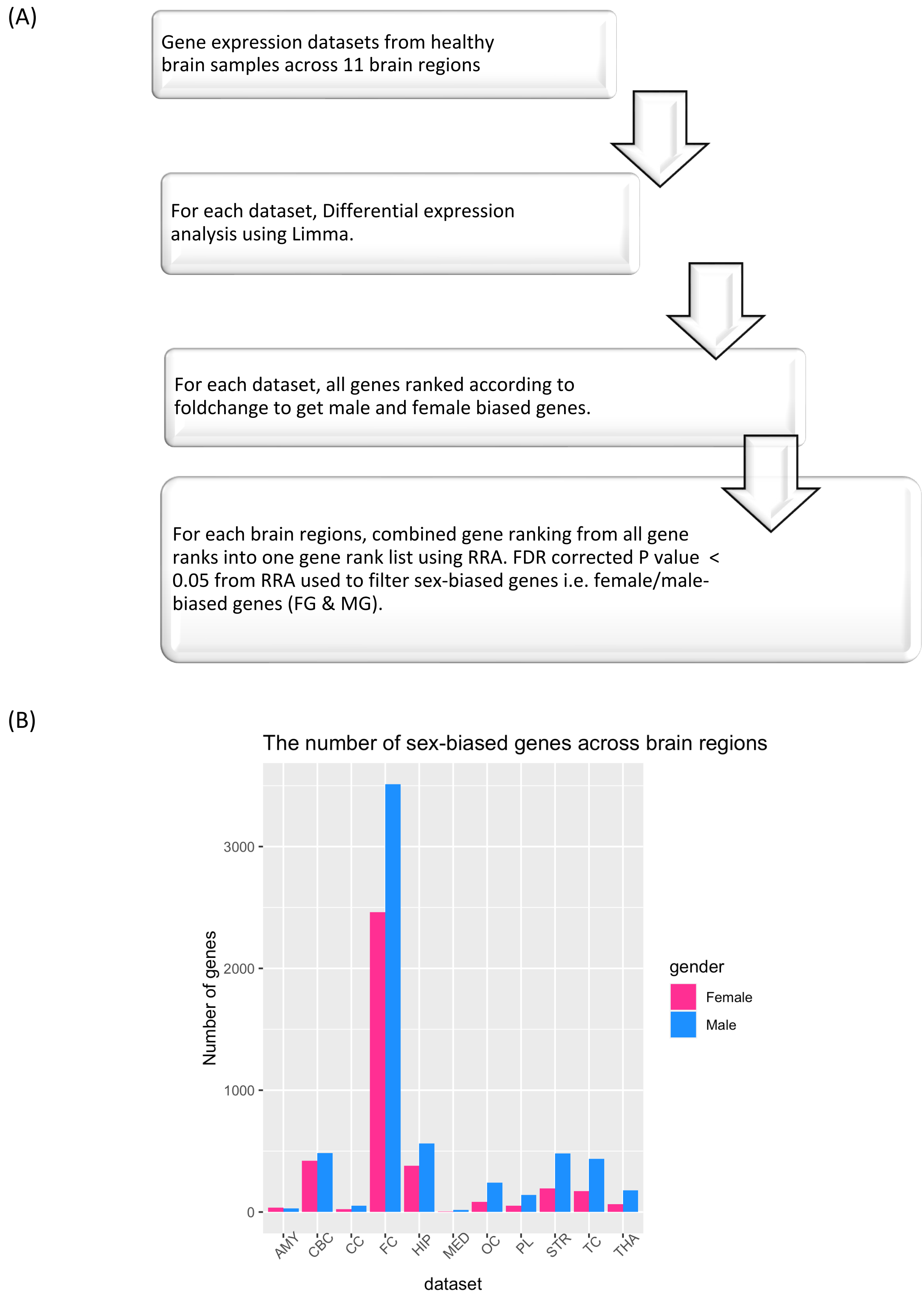

Fig S27. (A) Schematic diagram of additional workflow to generate sex-biased gene list. (B) The number of sex-biased genes from the additional workflow (S27A).

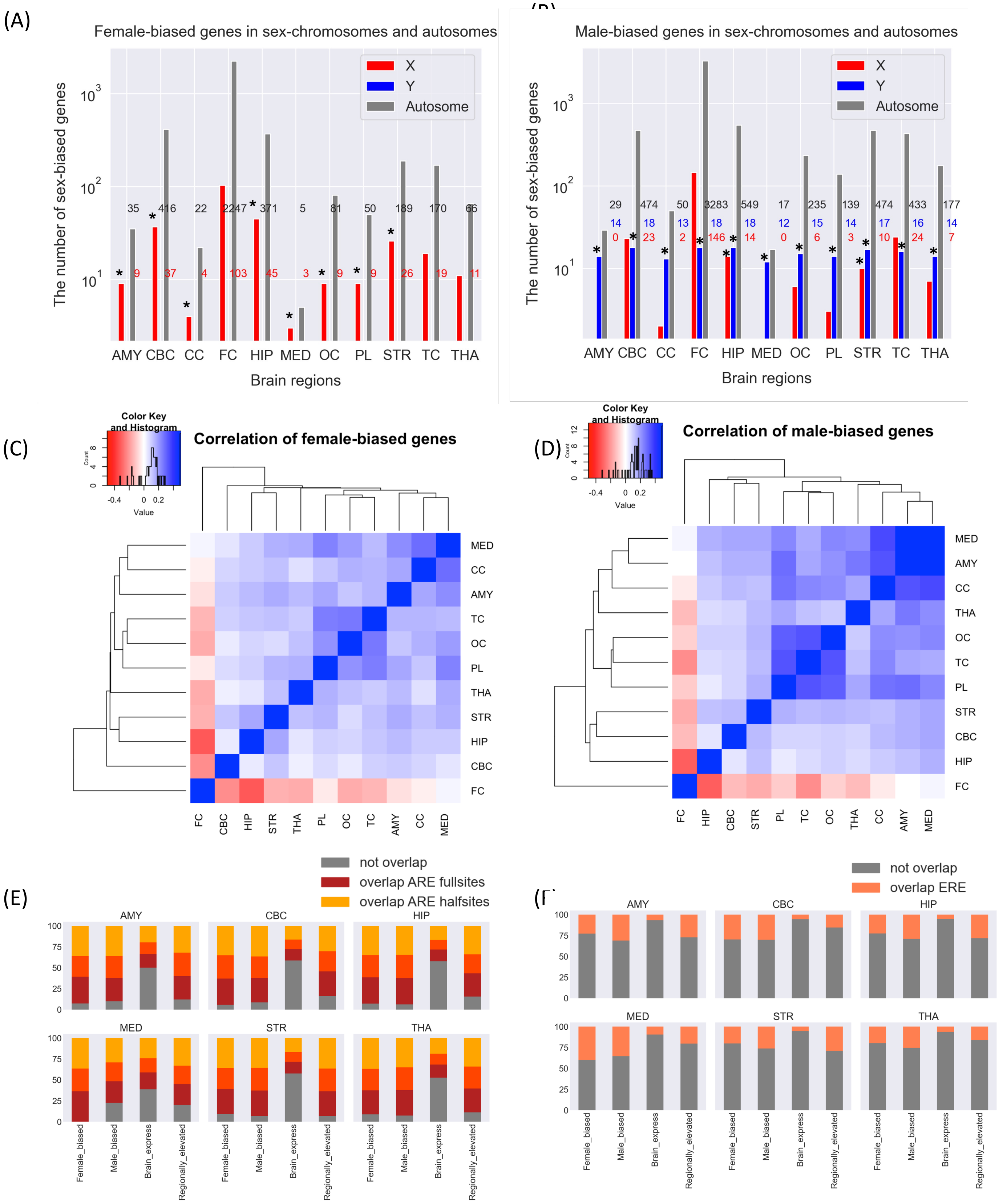

Fig S28. (A) The number of female-biased genes across brain regions using additional workflow (B) The number of male-biased genes across brain regions using additional workflow. (C) The correlation heatmap of female-biased genes using additional workflow. (D) The correlation heatmap of male-biased genes using additional workflow. (E) The percentage of overlap between Androgen receptor element (ARE) genes and sex-biased genes, brain expressed genes, and brain regionally elevated genes using additional workflow. (F) The percentage of overlap between Estrogen receptor element (ERE) genes and sex-biased genes. brain expressed genes and brain regionally elevated genes using additional workflow.

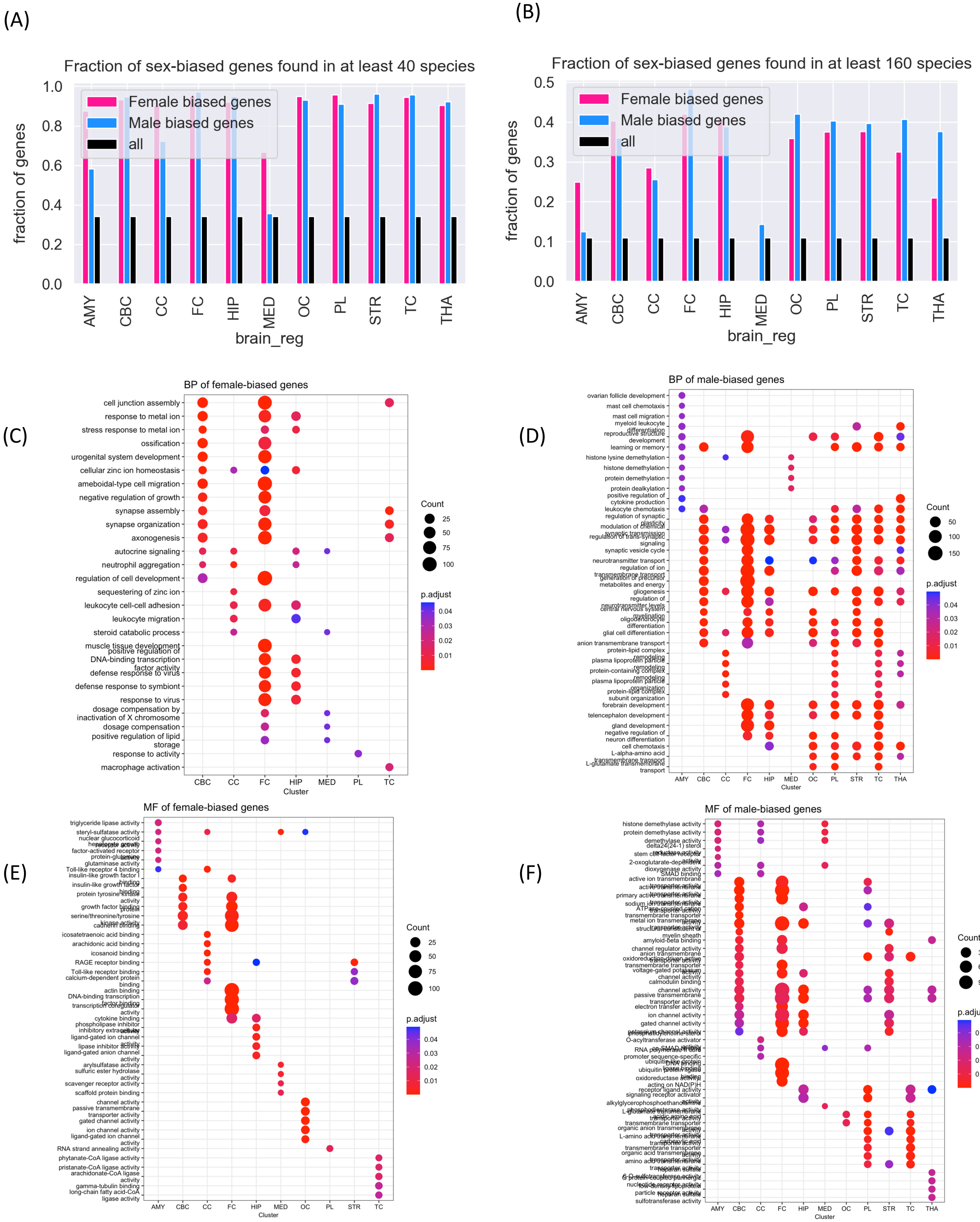

Fig S29. (A) Fraction of sex-biased genes found at least in 40 primates using additional workflow. (B) Fraction of sex-biased genes found at least in 160 primates using additional workflow. (C) Gene ontology enrichment analysis of female-biased genes for biological process using additional workflow. (D) Gene ontology enrichment analysis of male-biased genes for biological process using additional workflow. (E) Gene ontology enrichment analysis of female-biased genes for molecular function using additional workflow. (F) Gene ontology enrichment analysis of male-biased genes for molecular function using additional workflow.

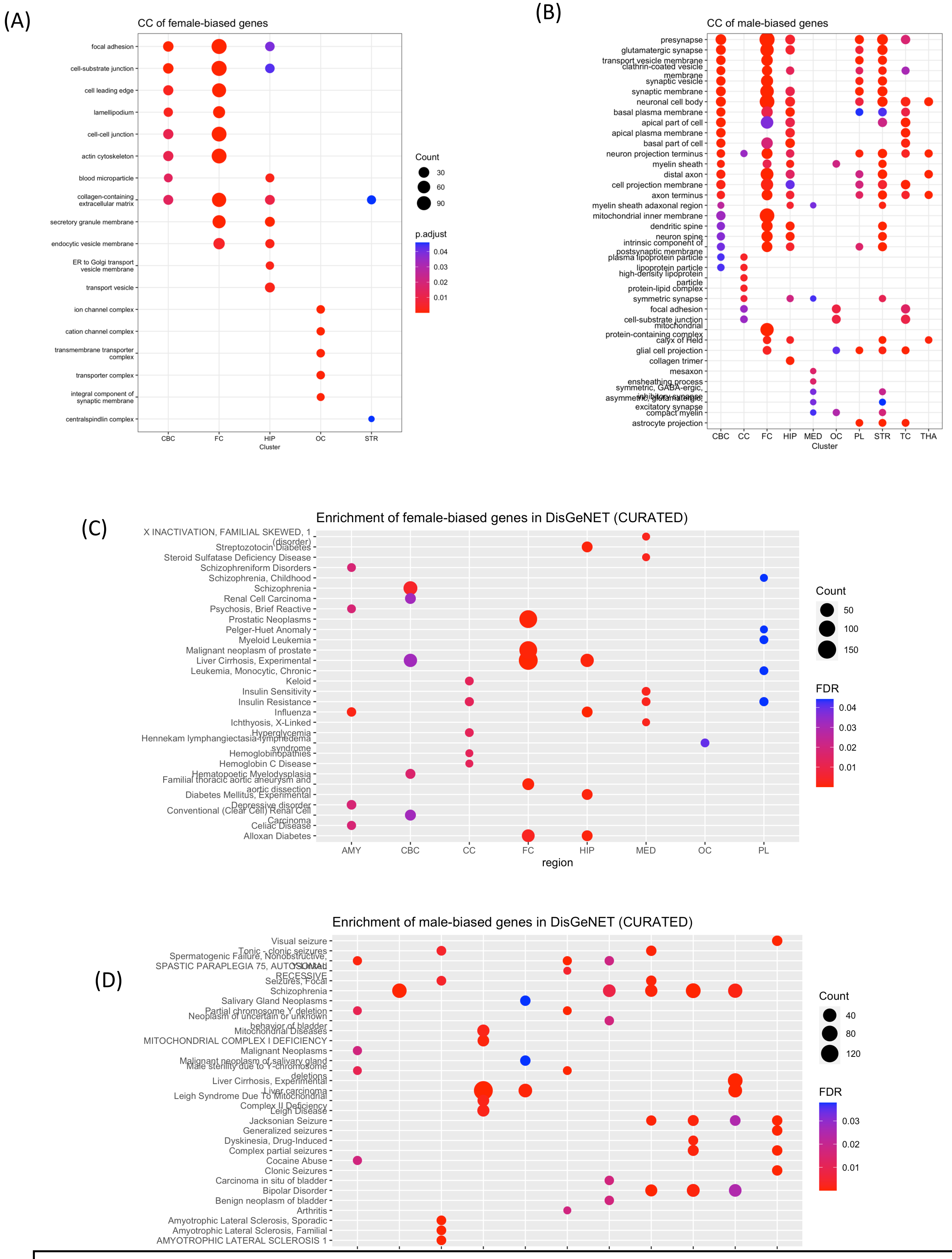

Fig S30. (A) Gene ontology enrichment analysis of female-biased genes for cellular composition using additional workflow. (B) Gene ontology enrichment analysis of male-biased genes for molecular cellular composition using additional workflow. (C) DisGeNet (CURATED) enrichment of female-biased genes using additional workflow. (D)) DisGeNet (CURATED) enrichment of male-biased genes using additional workflow.

(A)

DAVID Bioinformatics Resources
Laboratory of Human Retrovirology and Immunoinformatics (LHRI)

Functional Annotation Chart

Help and Manual

Current Gene List: F_THA_ID
Current Background: background_THA_ID
199 DAVID IDs
⊞ **Options**

Rerun Using Options | Create Sublist

570 chart records

Download File

Sublist	Category	Term	RT	Genes	Count	%	P-Value	Benjamini
☐	GOTERM_BP_ALL	central nervous system development	RT		33	16.6	6.4E-8	1.9E-4
☐	GOTERM_BP_ALL	single-multicellular organism process	RT		104	52.3	9.2E-8	1.9E-4
☐	GOTERM_BP_ALL	telencephalon development	RT		16	8.0	2.0E-7	2.8E-4
☐	GOTERM_BP_ALL	regulation of developmental process	RT		57	28.6	5.5E-7	5.7E-4
☐	GOTERM_BP_ALL	forebrain development	RT		18	9.0	1.0E-6	8.3E-4
☐	GOTERM_BP_ALL	nervous system development	RT		52	26.1	1.3E-6	8.3E-4
☐	GOTERM_BP_ALL	regulation of multicellular organismal process	RT		63	31.7	1.4E-6	8.3E-4
☐	GOTERM_BP_ALL	anatomical structure development	RT		94	47.2	2.2E-6	1.1E-3
☐	GOTERM_BP_ALL	regulation of multicellular organismal development	RT		47	23.6	2.4E-6	1.1E-3
☐	GOTERM_BP_ALL	developmental process	RT		99	49.7	4.1E-6	1.7E-3
☐	GOTERM_BP_ALL	regulation of nervous system development	RT		27	13.6	4.8E-6	1.8E-3
☐	GOTERM_BP_ALL	multicellular organism development	RT		86	43.2	5.2E-6	1.8E-3
☐	GOTERM_BP_ALL	tissue development	RT		44	22.1	5.6E-6	1.8E-3
☐	GOTERM_BP_ALL	positive regulation of developmental process	RT		36	18.1	6.9E-6	2.0E-3
☐	GOTERM_BP_ALL	localization	RT		97	48.7	8.2E-6	2.2E-3
☐	GOTERM_BP_ALL	positive regulation of multicellular organismal process	RT		41	20.6	8.4E-6	2.2E-3

(B)

DAVID Bioinformatics Resources
Laboratory of Human Retrovirology and Immunoinformatics (LHRI)

Functional Annotation Chart

Help and Manual

Current Gene List: M_THA_ID
Current Background: background_THA_ID
160 DAVID IDs
⊞ **Options**

Rerun Using Options | Create Sublist

591 chart records

Download File

Sublist	Category	Term	RT	Genes	Count	%	P-Value	Benjamini
☐	GOTERM_BP_ALL	regulation of immune system process	RT		38	23.8	8.2E-9	1.8E-5
☐	GOTERM_BP_ALL	multicellular organismal process	RT		100	62.5	1.2E-8	1.8E-5
☐	GOTERM_BP_ALL	single-multicellular organism process	RT		89	55.6	1.4E-8	1.8E-5
☐	GOTERM_BP_ALL	nervous system development	RT		48	30.0	3.6E-8	3.0E-5
☐	GOTERM_BP_ALL	cell-cell signaling	RT		37	23.1	3.9E-8	3.0E-5
☐	GOTERM_BP_ALL	anterograde trans-synaptic signaling	RT		23	14.4	7.1E-8	4.0E-5
☐	GOTERM_BP_ALL	chemical synaptic transmission	RT		23	14.4	7.1E-8	4.0E-5
☐	GOTERM_BP_ALL	trans-synaptic signaling	RT		23	14.4	9.1E-8	4.4E-5
☐	GOTERM_BP_ALL	synaptic signaling	RT		23	14.4	1.3E-7	5.6E-5
☐	GOTERM_BP_ALL	positive regulation of immune system process	RT		29	18.1	1.8E-7	6.8E-5
☐	GOTERM_BP_ALL	immune system process	RT		51	31.9	1.9E-7	6.8E-5
☐	GOTERM_BP_ALL	system development	RT		72	45.0	2.8E-7	9.2E-5
☐	GOTERM_BP_ALL	multicellular organism development	RT		75	46.9	4.4E-7	1.3E-4

Fig S31. Gene ontology analysis of output from DAVID (Database for Annotation, Visualization, and Integrated Discovery) for biological process. (A) The top GO terms in female-biased genes in THA region with background genes of THA gene expression data. (B) The top GO terms in male-biased genes in THA region with background genes of THA gene expression data.

References

1. Kolde, R., Laur, S., Adler, P., Vilo, J.: Robust rank aggregation for gene list integration and meta-analysis. Bioinformatics **28**(4), 573–580 (2012)
2. Chen, C.Y., Shi, W., Balaton, B.P., Matthews, A.M., Li, Y., Arenillas, D.J., Mathelier, A., Itoh, M., Kawaji, H., Lassmann, T., Hayashizaki, Y., Carninci, P., Forrest, A.R., Brown, C.J., Wasserman, W.W.: YY1 binding association with sex-biased transcription revealed through X-linked transcript levels and allelic binding analyses. Sci Rep **6**, 37324 (2016)
3. McKenzie, A.T., Wang, M., Hauberg, M.E., Fullard, J.F., Kozlenkov, A., Keenan, A., Hurd, Y.L., Dracheva, S., Casaccia, P., Roussos, P., Zhang, B.: Brain Cell Type Specific Gene Expression and Co-expression Network Architectures. Sci Rep **8**(1), 8868 (2018)
